# Efficacy of house dust mite allergen immunotherapy in allergic rhinitis: a network meta-analysis based on component-resolved classification

**DOI:** 10.3389/fimmu.2026.1947642

**Published:** 2026-09-03

**Authors:** Shuo Guo, Kun Wang, Yawen Shi, Fei Hong, Xingyu Zhang, Dahui Zha, Yisen Liu

**Affiliations:** 1 Department of Otolaryngology, Head and Neck Surgery, Hefei Third People’s Hospital, Hefei, Anhui, China; 2 School of Food and Biological Engineering, Hefei University of Technology, Hefei, Anhui, China; 3 Department of Otorhinolaryngology, The First Affiliated Hospital with Nanjing Medical University, Nanjing, Jiangsu, China

**Keywords:** allergen immunotherapy, allergic rhinitis, component-resolved classification, house dust mite, network meta-analysis, precision medicine, subcutaneous immunotherapy, sublingual immunotherapy

## Abstract

**Background:**

House dust mite allergen immunotherapy (HDM-AIT) trials in allergic rhinitis show substantial efficacy heterogeneity, traditionally evaluated by administration route. Whether clinical efficacy differs among HDM-AIT preparations with different allergen compositional breadth remains unclear.

**Objective:**

To compare HDM-AIT efficacy using a component-resolved framework that stratifies preparations by allergen compositional breadth.

**Methods:**

We searched PubMed, Embase, and CENTRAL for double-blind, placebo-controlled randomized trials of HDM-AIT lasting at least 12 months. Interventions were classified as comprehensive-component subcutaneous immunotherapy (CC-SCIT), major-component-dominant subcutaneous immunotherapy (MCD-SCIT), or major-component-dominant sublingual immunotherapy tablet (MCD-SLIT-tablet) using a prespecified component-resolved framework in which the breadth of treatment-induced component-specific IgG4 responses served as the primary node-defining criterion, with component-specific IgG and proteomic evidence used as supportive evidence. Frequentist pairwise and Bayesian network meta-analyses were performed. The primary outcome was symptom score; secondary outcomes were medication score and combined symptom and medication score.

**Results:**

Sixteen trials involving 7,329 participants were included. For symptom score, CC-SCIT showed the most favorable estimated treatment effect versus placebo (standardized mean difference [SMD], −0.40; 95% credible interval [CrI], −0.73 to −0.11; surface under the cumulative ranking curve [SUCRA], 82.6%), while MCD-SLIT-tablet showed a more precise estimate supported by a larger evidence base (SMD, −0.30; 95% CrI, −0.41 to −0.20; SUCRA, 60.7%). For medication score, CC-SCIT (SMD, −0.40; 95% CrI, −0.81 to 0.00; SUCRA, 79.8%) and MCD-SCIT (SMD, −0.38; 95% CrI, −0.72 to −0.05; SUCRA, 78.5%) showed similar estimated effects, whereas MCD-SLIT-tablet showed a smaller but more precise effect (SMD, −0.15; 95% CrI, −0.28 to 0.00; SUCRA, 39.9%). For combined symptom and medication score, CC-SCIT showed the largest estimated effect versus placebo (SMD, −0.84; 95% CrI, −1.53 to −0.37; SUCRA, 99.5%). The network was star-shaped, and comparisons among non-placebo treatment nodes were indirect. Posterior rank distributions also indicated uncertainty in the relative ordering of the treatment nodes, particularly those supported by fewer trials.

**Conclusion:**

Reported immunologic and compositional profiles of HDM-AIT preparations were associated with variability in trial-level efficacy estimates. These findings support product-level molecular characterization and direct comparative studies as priorities for future precision AIT.

**Systematic review registration:**

https://www.crd.york.ac.uk/PROSPERO/view/, CRD420261307571.

## 1 Introduction

House dust mite (HDM)–induced allergic diseases, including allergic rhinitis (AR), impose a substantial global burden and affect hundreds of millions of individuals worldwide (1). Allergen immunotherapy (AIT) remains the only causal treatment with disease-modifying potential, capable of redirecting allergen-specific immune responses and inducing sustained tolerance after treatment cessation (2). However, despite its long history of clinical use, the magnitude of benefit reported in HDM allergen immunotherapy (HDM-AIT) trials varies considerably across studies (3). This heterogeneity has traditionally been interpreted through the route of administration, most commonly subcutaneous immunotherapy (SCIT) versus sublingual immunotherapy (SLIT).

Route of administration, however, does not fully capture clinically relevant differences among HDM-AIT preparations. HDM extracts have historically been standardized according to total protein content and selected key allergens, particularly groups 1, 2, and 23 (4, 5). In addition, manufacturers use proprietary internal reference standards and report potency in product-specific units, such as IR, SQ-U, AU, or biological units, which are not directly interchangeable across preparations (4, 6). More importantly, HDM is not a single antigen but a complex allergen source. More than 40 Dermatophagoides allergens are listed by the WHO/IUIS Allergen Nomenclature database, and proteomic and immunochemical studies have shown substantial variation in the molecular composition and allergenic potency of commercial extracts derived from the same allergen source (7–10). These differences may arise from source materials, mite culture conditions, extraction procedures, purification steps, and formulation processes, leaving preparation-level allergen compositional breadth incompletely reflected by conventional standardization.

This product-level variability has direct clinical and methodological implications. Contemporary guidelines and position statements emphasize that evidence generated for one AIT preparation should not be automatically extrapolated to another, supporting a product-specific rather than class-wide approach to efficacy assessment (10–13). In parallel, component-resolved diagnostics have shown that patients with HDM allergy may be sensitized not only to major allergens such as Der p 1, Der p 2, and Der p 23, but also to intermediate and minor allergens, including Der p 4, Der p 5, Der p 7, and Der p 21 (14–18). These observations have prompted increasing interest in aligning the molecular composition of AIT preparations with the individual patient’s IgE sensitization profile, consistent with the broader precision medicine paradigm in allergic disease (19, 20). Therefore, differences in reported allergen composition and immunologic profiles of HDM-AIT preparations may represent one of several potential sources of variability in trial-level outcomes.

Existing meta-analyses and network meta-analyses (NMAs) have generally grouped HDM-AIT interventions by administration route or formulation type, thereby aggregating molecularly distinct preparations into broad intervention nodes (3, 21–23). This approach is clinically intuitive but may obscure product-level differences in allergen coverage and limit interpretation of between-study heterogeneity. To our knowledge, no previous NMA has reconstructed the HDM-AIT evidence network according to preparation allergen compositional breadth. Importantly, this framework does not presume that broader extracts are universally superior; rather, it treats compositional breadth as an explicit treatment-defining variable that can be tested within evidence synthesis. In this study, we developed a component-resolved classification framework and stratified interventions into comprehensive-component subcutaneous immunotherapy (CC-SCIT), major-component-dominant subcutaneous immunotherapy (MCD-SCIT), and major-component-dominant sublingual tablet immunotherapy (MCD-SLIT-tablet) nodes. Using both frequentist pairwise meta-analysis and Bayesian NMA, we aimed to compare HDM-AIT efficacy across randomized trials and to explore whether reported differences in immunologic and compositional characteristics of preparations are associated with variability in observed treatment effects.

## 2 Materials and methods

This systematic review and network meta-analysis followed the PRISMA-NMA guideline and was prospectively registered in PROSPERO (CRD420261307571). The protocol defined the clinical question, eligibility criteria, outcomes, and planned evidence synthesis before completion of the review.

The review question was structured according to PICOS. The population comprised patients with moderate-to-severe HDM-induced allergic rhinitis or trials of HDM-induced asthma with prespecified, extractable rhinitis outcomes. Eligible interventions were standardized HDM SCIT or SLIT-tablet preparations; the comparator was placebo. Outcomes were symptom score, medication score, and combined symptom and medication score. Eligible study designs were double-blind, placebo-controlled randomized trials with at least 12 months of treatment.

### 2.1 Search strategy and eligibility criteria

PubMed, Embase, and the Cochrane Central Register of Controlled Trials (CENTRAL) were searched from inception to February 20, 2026, using terms for house dust mite, allergic rhinitis/rhinoconjunctivitis, and allergen immunotherapy. Full strategies are provided in **Supplementary Table 1** in the **Supplementary Material**. Reference lists of included trials and relevant recent reviews were screened manually. Records were deduplicated in Rayyan.

Eligible studies were double-blind, placebo-controlled randomized controlled trials evaluating house dust mite allergen immunotherapy (HDM-AIT) for at least 12 months. Participants had moderate-to-severe HDM-induced allergic rhinitis, or HDM-induced asthma with prespecified, extractable allergic rhinitis outcomes. Eligible interventions were standardized subcutaneous immunotherapy (SCIT) or sublingual immunotherapy (SLIT) tablet formulations containing HDM extract. Liquid SLIT formulations were excluded because heterogeneity in product-level allergen composition, dosing units, and standardization procedures precluded reliable component-resolved node assignment. Mixed-allergen trials without isolatable HDM-specific effects and studies with unavailable outcome data after author contact were also excluded.

### 2.2 Study selection, data extraction, and outcomes

Two investigators independently screened titles and abstracts, reviewed full texts, and extracted data using a standardized Excel template; disagreements were resolved by consensus or consultation with a third reviewer. Extracted variables included study design, country, recruitment setting, sample size, participant age and clinical phenotype, baseline severity, intervention formulation and dose-unit system, treatment duration, outcome definition, assessment time point, and numerical data required for effect-size calculation. Preparation and dose information was also used to link each trial arm to the external component-resolved evidence used for node assignment.

The primary outcome was symptom score (SS). Secondary outcomes were medication score (MS) and combined symptom and medication score (CSMS), extracted according to each trial’s original definitions. Graphical data were digitized using metaDigitise in R 4.4.2. Means and standard deviations were estimated from medians and interquartile ranges using the methods of Luo et al. (24) and Wan et al. (25).

### 2.3 Component-resolved preparation classification

Before analysis, AIT treatment arms were assigned to predefined exploratory component-resolved nodes according to reported immunologic and compositional characteristics of house dust mite immunotherapy preparations in previously published studies (16, 17, 26–34). Node assignment was based exclusively on external component-resolved immunologic and compositional evidence. Clinical efficacy data from the included trials, including symptom score, medication score, combined symptom and medication score, and their corresponding effect estimates, were not used as criteria for treatment-node assignment. The original assignment was completed without reference to the extracted efficacy estimates, although it was not conducted under a formally documented outcome-masking procedure.

Comprehensive-component SCIT (CC-SCIT) was defined as preparations for which prior immunologic studies reported treatment-induced component-specific IgG4 responses extending beyond the dominant allergens Der p 1, Der p 2, and Der p 23 to multiple additional HDM components, particularly Der p 5, Der p 7, and Der p 21. Der p 4 was considered supportive when available but was not required for classification, because it was less consistently reported across studies. MCD groups were defined as those for which published immunologic evidence indicated that treatment-associated immune responses were predominantly directed toward major allergens, with limited or absent reported expansion toward intermediate components such as Der p 5, Der p 7, and Der p 21.

Operationally, node assignment followed a four-step algorithm: first, trial arms were linked to the corresponding preparation or preparation family using manufacturer, formulation, source material, standardization system, and other available product information; second, treatment-induced component-specific IgG4 response breadth was assessed as the primary evidence for treatment-node assignment; third, preparations were assigned to the CC or MCD category according to the definitions above; and fourth, component-specific IgG, proteomic, and product-level data were used as supportive evidence where available to support and contextualize the IgG4-based assignment rather than as co-equal node-defining criteria. These supporting data were also considered when assessing assignment certainty, particularly when the available IgG4 evidence was indirect or incomplete. Historical serum IgE sensitization profiles were not used for classification.

To assess reproducibility, two investigators independently reassessed all treatment-node assignments using this four-step algorithm while masked to the extracted clinical efficacy estimates. Inter-rater agreement was assessed using Cohen’s κ, and disagreements were resolved by consensus or consultation with a third investigator. This classification framework was applied only to trial arms included in this review.

The classification hierarchy intentionally separated direct analytical evidence from functional evidence. Quantitative or qualitative LC-MS/MS and other proteomic data were treated as evidence of reported preparation composition. Published product, source-material, or manufacturing information was used to support linkage within a preparation group. Treatment-induced component-specific IgG4 response breadth served as the prespecified node-defining reference standard. Component-specific IgG and proteomic or product-level data were used as supportive evidence where applicable. The operational term “comprehensive-component” therefore denoted broader reported component-level coverage or immunogenicity and did not imply that every WHO/IUIS-listed HDM allergen was present. Evidence strength was not uniform across preparation groups. Assignments supported by concordant proteomic and treatment-induced antibody data were considered more direct, whereas assignments relying on evidence from the same preparation family or on discordant historical and later immunogenicity observations were retained with lower certainty.

### 2.4 Risk of bias

Two investigators independently assessed randomized trials using the Cochrane Risk of Bias 2 tool (35). Five domains were evaluated: randomization, deviations from intended interventions, missing outcome data, outcome measurement, and selection of the reported result. Overall judgments were low risk, some concerns, or high risk.

### 2.5 Statistical analysis

Because outcome scales varied across trials, treatment effects were expressed as standardized mean differences (SMDs) with Hedges’ g small-sample correction; negative values favored treatment. Change-from-baseline values were used when available. Direct placebo-controlled evidence for each node was first summarized using pairwise random-effects meta-analysis with restricted maximum likelihood estimation in the R meta package. Estimates were reported with 95% confidence intervals (CIs), and heterogeneity was described using Cochran’s Q, I², and τ². These analyses were used to show the observed evidence within each node before indirect network comparisons were considered.

Bayesian random-effects network meta-analysis was performed with gemtc and JAGS in R (36). The network included placebo, CC-SCIT, MCD-SCIT, and MCD-SLIT-tablet. Four Markov chain Monte Carlo chains were run for 100,000 iterations after 50,000 burn-in iterations; convergence was assessed with the Gelman–Rubin statistic. Treatment ranking was summarized using the SUCRA (37). Ranking uncertainty was additionally characterized by the posterior mean rank, the probability of ranking first, and the 95% credible rank interval derived from the posterior rank distribution. Because the network was star-shaped and contained no closed loops, inconsistency could not be formally assessed; treatment comparisons were therefore interpreted as indirect and exploratory. Model fit was compared between fixed-effects and random-effects Bayesian models using the deviance information criterion.

Small-study effects were examined using comparison-adjusted funnel plots. Egger’s test was applied only when at least 10 studies contributed to an outcome (38). Because individual SCIT-versus-placebo comparisons included no more than four trials, the comparison-adjusted funnel plots and regression tests were considered exploratory. Sensitivity analyses included leave-one-out analyses, exclusion of trials published before 2005, and exclusion of Japanese studies with near-zero baseline MS to evaluate floor effects.

### 2.6 Ethics

This study used aggregated data from published trials and required no institutional review board approval or new informed consent.

## 3 Results

### 3.1 Study selection and trial characteristics

**Figure 1:**
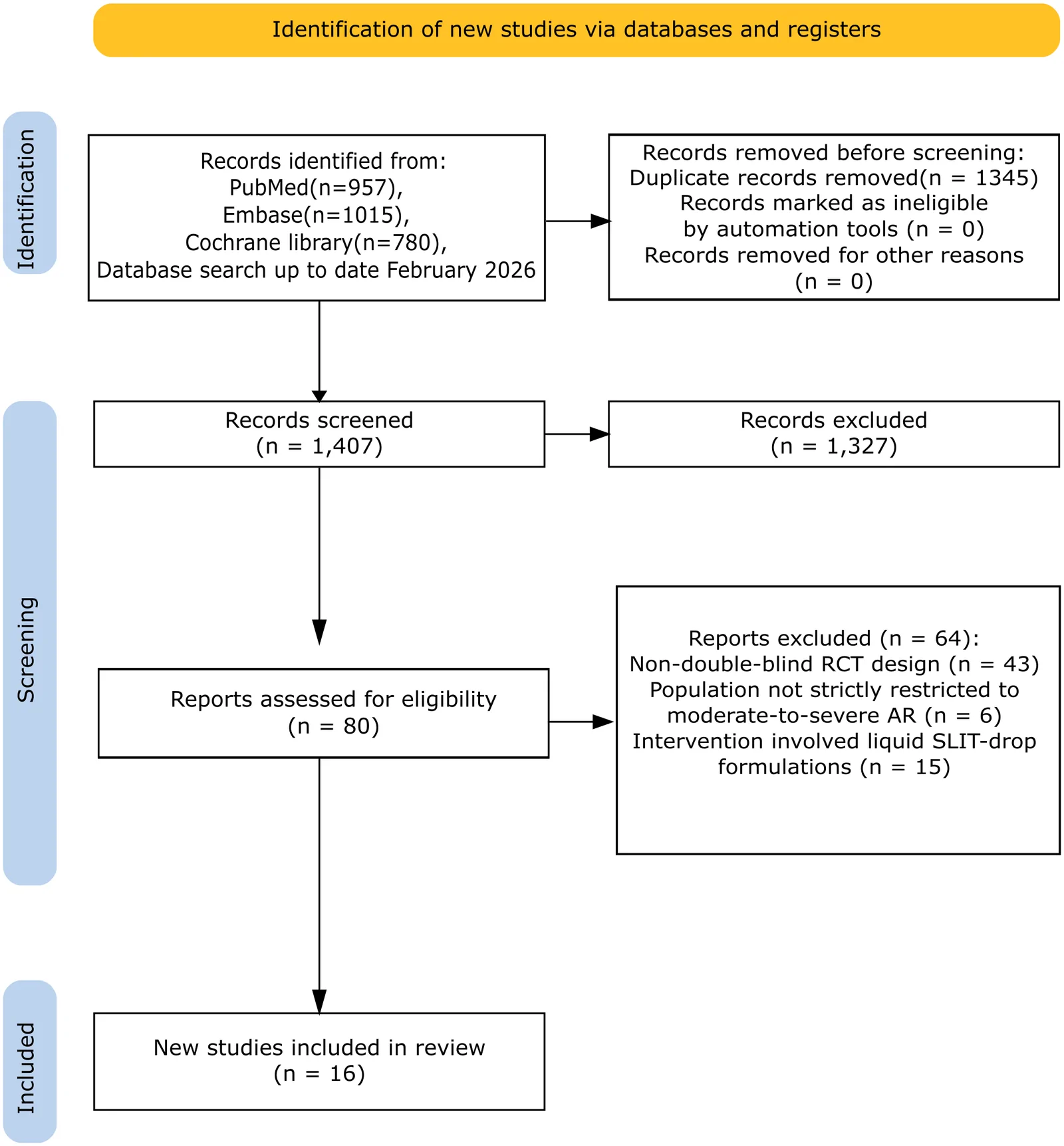
PRISMA flow diagram of study selection.

The database search identified 2,752 records: 957 from PubMed, 1,015 from Embase, and 780 from CENTRAL. After removal of 1,345 duplicates, 1,407 records underwent title and abstract screening, of which 80 were assessed in full text. Sixty-four reports were excluded because of non-double-blind randomized controlled trial design (n = 43), liquid SLIT-drop formulations (n = 15), or populations not restricted to moderate-to-severe allergic rhinitis (n = 6). Sixteen double-blind, placebo-controlled randomized trials were included in the final analysis (**Figure 1**).

The included trials were published between 1990 and 2025 and enrolled 7,329 participants. Treatment duration ranged from 12 to 24 months. According to the component-resolved classification framework, interventions were assigned to three nodes: comprehensive-component subcutaneous immunotherapy (CC-SCIT; 3 trials), major-component-dominant subcutaneous immunotherapy (MCD-SCIT; 4 trials), and major-component-dominant sublingual immunotherapy tablet (MCD-SLIT-tablet; 9 trials). Within the MCD-SLIT-tablet node, four trials evaluated 300 IR, three evaluated 12 SQ-HDM, and two evaluated 6 SQ-HDM. Independent reassessment showed 100% agreement in treatment-node assignment between the two investigators before consensus (Cohen’s κ = 1.00). Detailed study characteristics are summarized in **Table 1**.

**Table 1:** Characteristics of included double-blind randomized controlled trials.

Study (Year)	Country	Participants in each group		Baseline severity	Age	Treatment duration (months)	Outcome	Maintenance dose
		Active	Placebo					
CC-SCIT								
Yukselen 2012 (39)	Turkey	10	10	HDM-Asthma with AR^†^	10.9 ± 3.2	12	SS, MS	4000 TU q4w
Bozek 2017 (40)	Poland	29	26	Moderate-to-severe AR	68.1 ± 5.9	24	SS, CSMS	10000 AUeq q4w
Bachert 2025^††^ (41)	7 European countries	105	123	Moderate-to-severe AR	33 ± 11	12	SS, MS, CSMS	25000 AUeq q4w
MCD-SCIT								
McHugh 1990 (42)	United Kingdom	27	28	Moderate-to-severe AR	17-52	12	SS, MS	17000 BU q4w
Varney 2003 (43)	United Kingdom	15	13	Moderate-to-severe AR	33(19,48)	12	SS, MS	100000 SQ-U q4w
Xian 2019 (44)	China	26	14	Moderate-to-severe AR	21.1 ± 12.0	12	SS, MS, CSMS	100000 SQ-U q6w
Suratannon 2024 (45)	Thailand	96	34	Moderate-to-severe AR	30.5 ± 9.6	12	SS, MS, CSMS	500 AU q4w
MCD-SLIT-Tablet								
Bergmann 2014 (46)	7 European countries	141	153	Moderate-to-severe AR	29.0 ± 8.5	12	SS, MS, CSMS	300 IR qd
Demoly 2016 (47)	12 European countries	284	298	Moderate-to-severe AR	32.1 ± 10.6	12	SS, MS, CSMS	12 SQ qd
Nolte 2016 (48)	United States and Canada	566	620	Moderate-to-severe AR	35 ± 14	12	SS, MS, CSMS	12 SQ qd
Okamoto 2017 (49)	Japan	315	316	Moderate-to-severe AR	30.0 ± 11.8	12	SS, MS, CSMS	300 IR qd
Okubo 2017 (50)	Japan	285	285	Moderate-to-severe AR	27.2 ± 12.0	12	SS, MS, CSMS	6 SQ qd
Masuyama 2018 (51)	Japan	209	218	Moderate-to-severe AR	10.8 ± 2.9	12	SS, MS, CSMS	6 SQ qd
Okamoto 2019 (52)	Japan	205	217	Moderate-to-severe AR	10.3 ± 2.7	12	SS, MS, CSMS	300 IR qd
Demoly 2020 (53)	13 countries	586	676	Moderate-to-severe AR	29.5 ± 13.1	12	SS, MS, CSMS	300 IR qd
Schuster 2025 (54)	Europe and North America	693	706	Moderate-to-severe AR	8 ± 1.9	12	SS, MS, CSMS	12 SQ qd

AR, allergic rhinitis; AU, allergy units; AUeq, allergy units equivalent; BU, biological units; CC-SCIT, comprehensive-component subcutaneous immunotherapy; CSMS, combined symptom and medication score; HDM, house dust mite; IR, index of reactivity; MCD-SCIT, major-component-dominant subcutaneous immunotherapy; MCD-SLIT-tablet, major-component-dominant sublingual immunotherapy tablet; MS, medication score; q4w, every 4 weeks; q6w, every 6 weeks; qd, once daily; SCIT, subcutaneous immunotherapy; SLIT, sublingual immunotherapy; SQ, standardized quality; SQ-U, standardized quality units; SS, symptom score; TU, therapeutic units. Age is presented as mean ± SD, range, or median (range), as reported in the original studies. ^†^Trial enrolled patients with HDM-induced asthma but reported prespecified allergic rhinitis outcomes. ^††^Bachert 2025 data were extracted from the moderate-to-severe subgroup reported in the trial.

The evidence supporting node assignment is detailed in **Table 2**. Treatment-induced component-specific IgG4 response breadth served as the primary basis for classification, with component-specific IgG and proteomic evidence used for supportive characterization where applicable. Yukselen 2012, Bozek 2017, and Bachert 2025 were classified as CC-SCIT based on broad component-specific immunogenicity profiles extending beyond Der p 1, Der p 2, and Der p 23 to intermediate allergens, including Der p 5, Der p 7, and Der p 21. In contrast, MCD-SCIT and MCD-SLIT-tablet were characterized by responses predominantly directed toward Der p 1, Der p 2, and Der p 23, with limits toward Der p 5, Der p 7, and Der p 21. These distinct response patterns formed the basis of the component-resolved treatment nodes.

**Table 2:** Component-resolved evidence supporting treatment-node assignment.

Node	Included trials	Dose-unit pattern	Evidence supporting assignment
CC-SCIT	Yukselen 2012 (39)	4000 TU q4w	A multicenter study showed treatment-induced sIgG4 responses to all assessed HDM components, including Der p 5, Der p 7, and Der p 21, and LC-MS/MS confirmed major, mid-tier, and most minor allergens in the corresponding HDM AIT preparation group (27, 28, 34)
CC-SCIT	Bozek 2017 (40) Bachert 2025 (41)	10000 AUeq q4w 25000 AUeq q4w	A retrospective serum analysis from an earlier AIT trial (2001) reported IgG induction mainly to Der p 1 and Der p 2, without relevant increases to Der p 23, Der p 5, Der p 7, or Der p 21 (31). Later preclinical component-resolved immunogenicity data reported detectable IgG responses to Der p 5, Der p 7, and Der p 21 (29). Assigned to CC-SCIT with lower certainty.
MCD-SCIT	McHugh 1990 (42) Varney 2003 (43) Xian 2020 (44) Suratannon 2024 (45)	17000 BU q4w 100000 SQ-U q4w/q6w 500 AU q4w	Component-resolved monitoring showed treatment-induced IgG mainly to Der p 1/2 and Der p 23, but not to Der p 5, Der p 7, or Der p 21; efficacy was greater in patients sensitized only to Der p 1 and/or Der p 2 (16).
MCD-SLIT-tablet	Demoly 2016 (47) Nolte 2016 (48) Okubo 2017 (50) Masuyama 2018 (51) Schuster 2025 (54)	6 SQ qd 12 SQ qd	A study showed AIT increased total component-specific IgE and IgG4 levels but did not substantially broaden recognition beyond the pre-existing major-allergen pattern; intermediate-component IgG4 responses showed no meaningful treatment-induced expansion (32).
MCD-SLIT-tablet	Bergmann 2014 (46); Okamoto 2017 (49); Okamoto 2019 (52); Demoly 2020 (53)	300 IR qd	Component-resolved evidence showed IgG/IgG4 increases mainly to Der p 1, Der p 2, and Der p 23, whereas broad sIgG4 induction to Der p 5, Der p 7, and Der p 21 was not demonstrated (33).

Treatment-node assignment was based primarily on treatment-induced component-specific IgG4 response breadth. Component-specific IgG and quantitative proteomic or LC-MS/MS evidence were used as supportive evidence when available. Historical serum IgE profiles were not used for treatment-node assignment. AIT, allergen immunotherapy; AU, allergy units; AUeq, allergy units equivalent; BU, biological units; CC-SCIT, comprehensive-component subcutaneous immunotherapy; HDM, house dust mite; IR, index of reactivity; LC-MS/MS, liquid chromatography-coupled tandem mass spectrometry; MCD-SCIT, major-component-dominant subcutaneous immunotherapy; MCD-SLIT-tablet, major-component-dominant sublingual immunotherapy tablet; q4w, every 4 weeks; q6w, every 6 weeks; qd, once daily; SCIT, subcutaneous immunotherapy; sIgG4, specific IgG4; SLIT, sublingual immunotherapy; SQ, standardized quality; SQ-U, standardized quality units; TU, therapeutic units.

### 3.2 Network geometry and risk of bias

**Figure 2:**
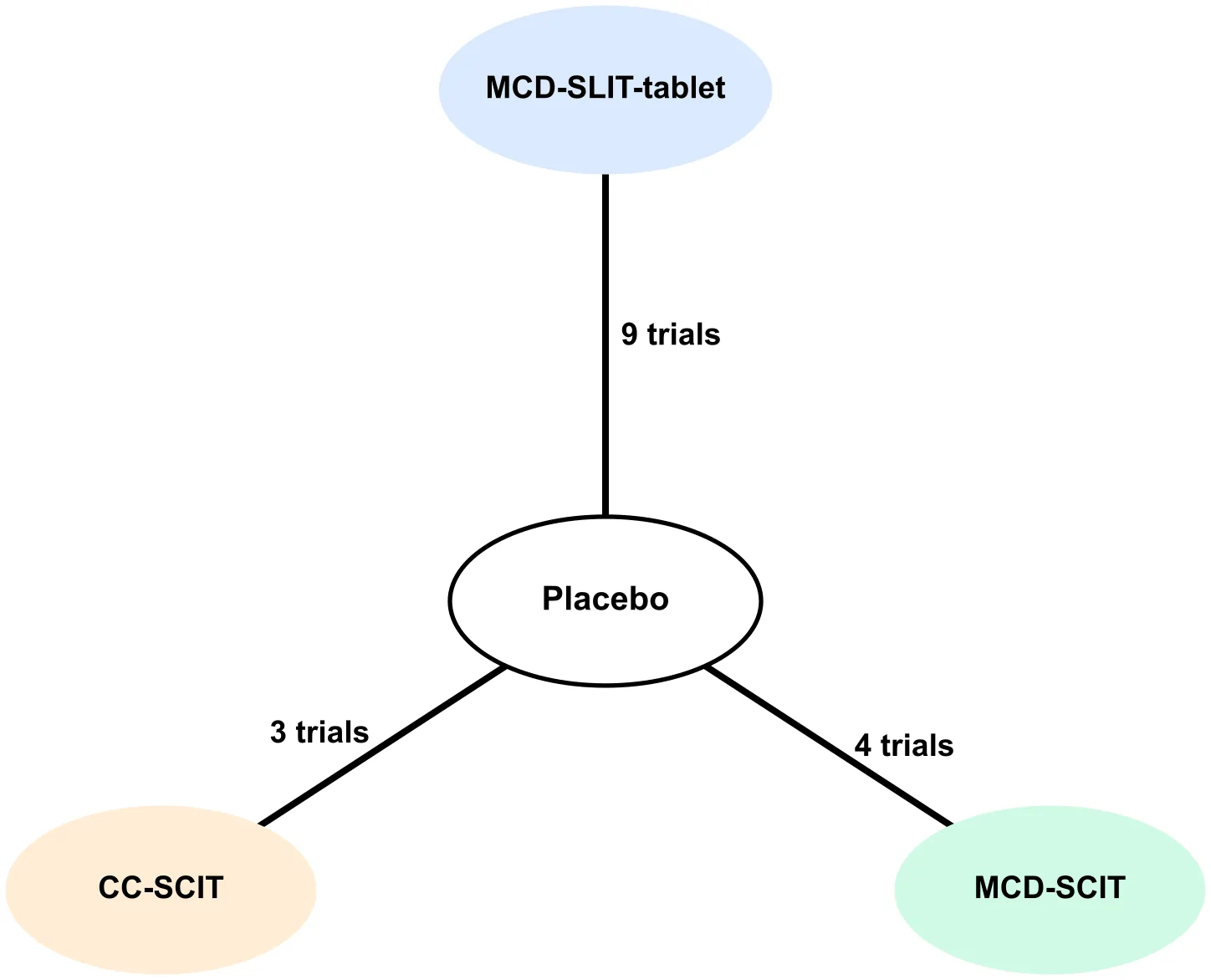
Network geometry of included treatment nodes.

The evidence network was star-shaped, with placebo as the sole common comparator and no direct head-to-head comparisons among non-placebo interventions (**Figure 2**). CC-SCIT was represented by 3 trials, MCD-SCIT by 4 trials, and MCD-SLIT-tablet by 9 trials. The precision of the evidence was correspondingly uneven: SCIT nodes were based on smaller samples, whereas the SLIT-tablet node included several large multicenter trials. Therefore, all comparisons between treatment nodes were indirect and dependent on cross-trial comparability.

Random-effects models provided lower DIC values than fixed-effect models for SS (30.89 vs 36.06), MS (28.50 vs 47.63), and CSMS (34.09 vs 43.90), supporting the primary use of random-effects estimates (**Supplementary Table 2** in the **Supplementary Material**).

Ten trials were judged to have low overall risk of bias, and six had some concerns (**Supplementary Figures 1**, **S2** in the **Supplementary Material**). Concerns were mainly related to selection of the reported result, with additional concerns due to missing outcome data in two trials and randomization in one trial. No trial was judged to be at high risk of bias.

### 3.3 Primary outcome: symptom score

For symptom score, the Bayesian random-effects model showed low-to-moderate network heterogeneity (τ = 0.110; 95% CrI, 0.02 to 0.27; network I² = 29%). In frequentist pairwise meta-analysis, CC-SCIT showed a significant benefit versus placebo (3 trials; 144 receiving CC-SCIT/159 receiving placebo; SMD, −0.49; 95% CI, −0.92 to −0.06; I² = 50%; τ² = 0.072; P = 0.137). MCD-SCIT showed a numerical but non-significant effect (4 trials; 164 receiving MCD-SCIT/89 receiving placebo; SMD, −0.35; 95% CI, −0.82 to 0.12; I² = 63%; τ² = 0.139; P = 0.055). MCD-SLIT-tablet also showed a significant benefit versus placebo (9 trials; 3,284 receiving MCD-SLIT-tablet/3,489 receiving placebo; SMD, −0.29; 95% CI, −0.37 to −0.22; I² = 58%; τ² = 0.008; P = 0.018) (**Supplementary Figure 3A**).

**Figure 3:**
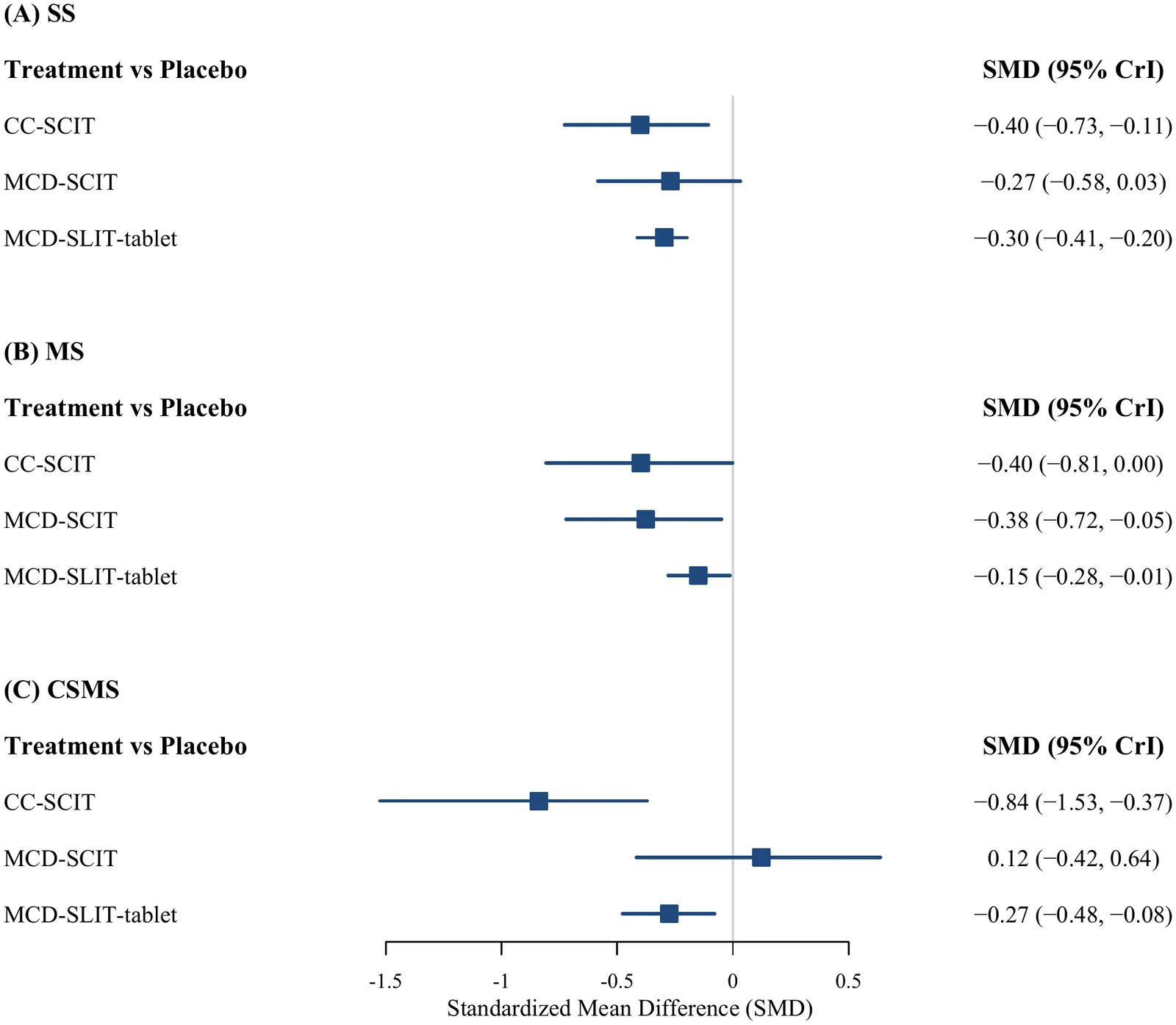
Bayesian network estimates versus placebo. Forest plots showing standardized mean differences (SMDs) with 95% credible intervals (CrIs) for symptom score **(A)**, medication score **(B)**, and combined symptom and medication score **(C)**.

**Figure 4:**
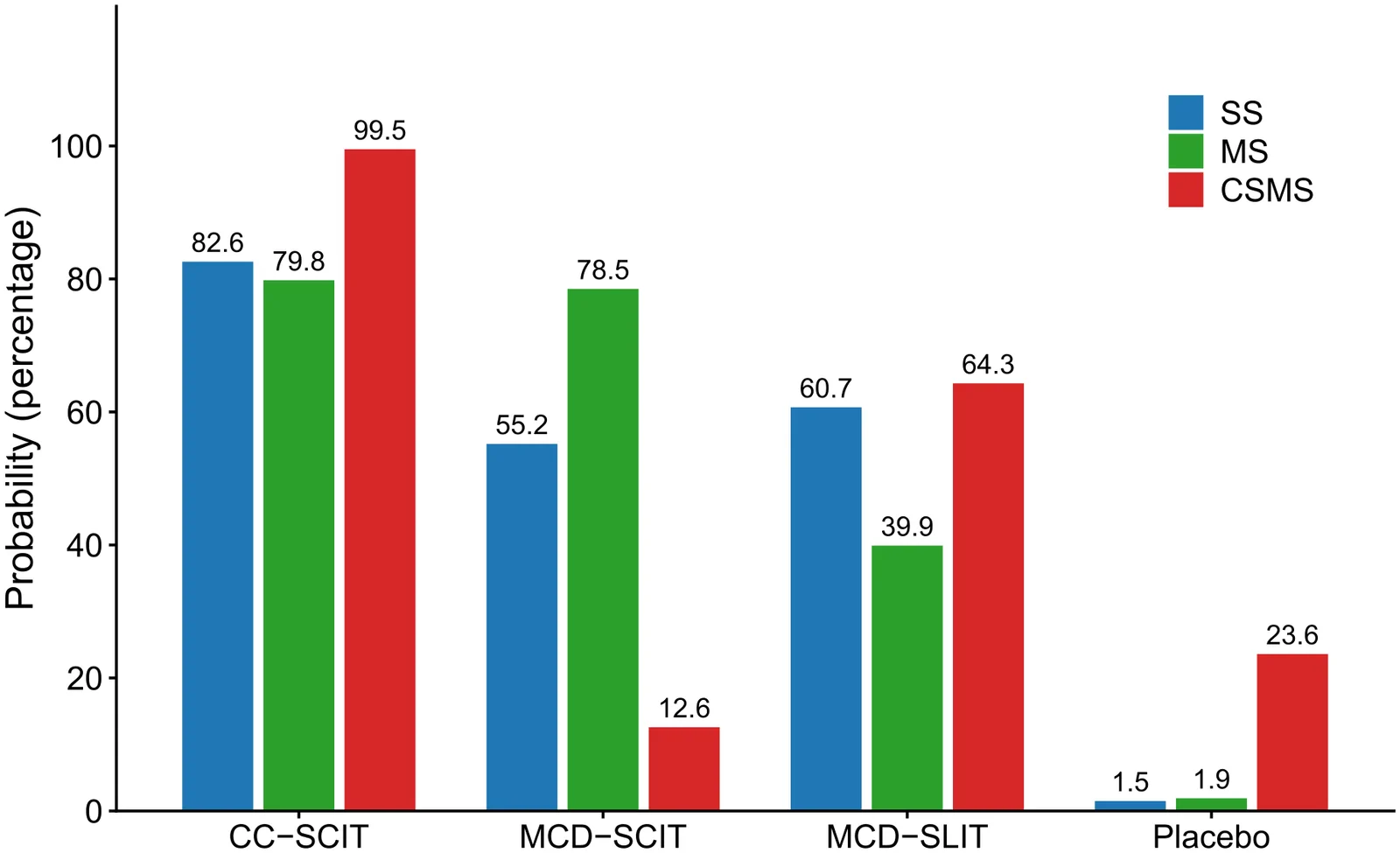
Treatment ranking according to SUCRA. Surface under the cumulative ranking curve (SUCRA) probabilities for symptom score, medication score, and combined symptom and medication score across all treatment nodes.

Bayesian network estimates were consistent with the pairwise analyses. Compared with placebo, the SMD was −0.40 for CC-SCIT (95% CrI, −0.73 to −0.11), −0.27 for MCD-SCIT (95% CrI, −0.58 to 0.03), and −0.30 for MCD-SLIT-tablet (95% CrI, −0.41 to −0.20) (**Figure 3A**). SUCRA rankings were highest for CC-SCIT (82.6%), followed by MCD-SLIT-tablet (60.7%), MCD-SCIT (55.2%), and placebo (1.5%) (**Figure 4**). For symptom score, CC-SCIT had a posterior mean rank of 1.52 (95% credible rank interval, 1–3) and a 63.3% probability of ranking first. Posterior ranking measures for all treatments and outcomes are reported in **Supplementary Table 4** in the **Supplementary Material**.

### 3.4 Secondary outcomes: medication score and combined symptom and medication score

For medication score, the Bayesian model showed low overall network heterogeneity (τ = 0.160; 95% CrI, 0.07 to 0.34; network I² = 22%). Pairwise meta-analysis favored CC-SCIT versus placebo (2 trials; 115 receiving CC-SCIT/133 receiving placebo; SMD, −0.37; 95% CI, −0.62 to −0.12; I² = 0%; τ² = 0.000; P = 0.494). MCD-SCIT showed a non-significant pairwise effect with substantial heterogeneity (4 trials; 164 receiving MCD-SCIT/89 receiving placebo; SMD, −0.47; 95% CI, −1.00 to 0.07; I² = 71%; τ² = 0.209; P = 0.013). MCD-SLIT-tablet showed a smaller but significant reduction in medication score (9 trials; 3,284 receiving MCD-SLIT-tablet/3,489 receiving placebo; SMD, −0.15; 95% CI, −0.25 to −0.06; I² = 72%; τ² = 0.014; P < 0.001) (**Supplementary Figure 3B**).

Bayesian estimates versus placebo were −0.40 for CC-SCIT (95% CrI, −0.81 to −0.01), −0.38 for MCD-SCIT (95% CrI, −0.72 to −0.05), and −0.15 for MCD-SLIT-tablet (95% CrI, −0.28 to 0.00) (**Figure 3B**). SUCRA rankings were similar for CC-SCIT and MCD-SCIT (79.8% and 78.5%, respectively), followed by MCD-SLIT-tablet (39.9%) and placebo (1.9%) (**Figure 4**).

For combined symptom and medication score, network heterogeneity was moderate (τ = 0.210; 95% CrI, 0.03 to 0.57; network I² = 46%). In pairwise meta-analysis, CC-SCIT showed a large but imprecise effect because of extreme heterogeneity (2 trials; 134 receiving CC-SCIT/149 receiving placebo; SMD, −1.23; 95% CI, −3.07 to 0.61; I² = 96%; τ² = 1.697; P < 0.001). MCD-SCIT showed no significant benefit (2 trials; 122 receiving MCD-SCIT/48 receiving placebo; SMD, 0.15; 95% CI, −0.18 to 0.49; I² = 0%; τ² = 0.000; P = 0.356). MCD-SLIT-tablet showed a significant reduction with low-to-moderate heterogeneity (9 trials; 3,284 receiving MCD-SLIT-tablet/3,489 receiving placebo; SMD, −0.26; 95% CI, −0.32 to −0.20; I² = 34%; τ² = 0.003; P = 0.179) (**Supplementary Figure 3C**).

Bayesian estimates versus placebo were −0.84 for CC-SCIT (95% CrI, −1.53 to −0.37), 0.12 for MCD-SCIT (95% CrI, −0.42 to 0.64), and −0.27 for MCD-SLIT-tablet (95% CrI, −0.48 to −0.08) (**Figure 3C**). SUCRA values were 99.5% for CC-SCIT, 64.3% for MCD-SLIT-tablet, 23.6% for placebo, and 12.6% for MCD-SCIT (**Figure 4**).

### 3.5 Indirect comparisons and sensitivity analyses

**Figure 5:**
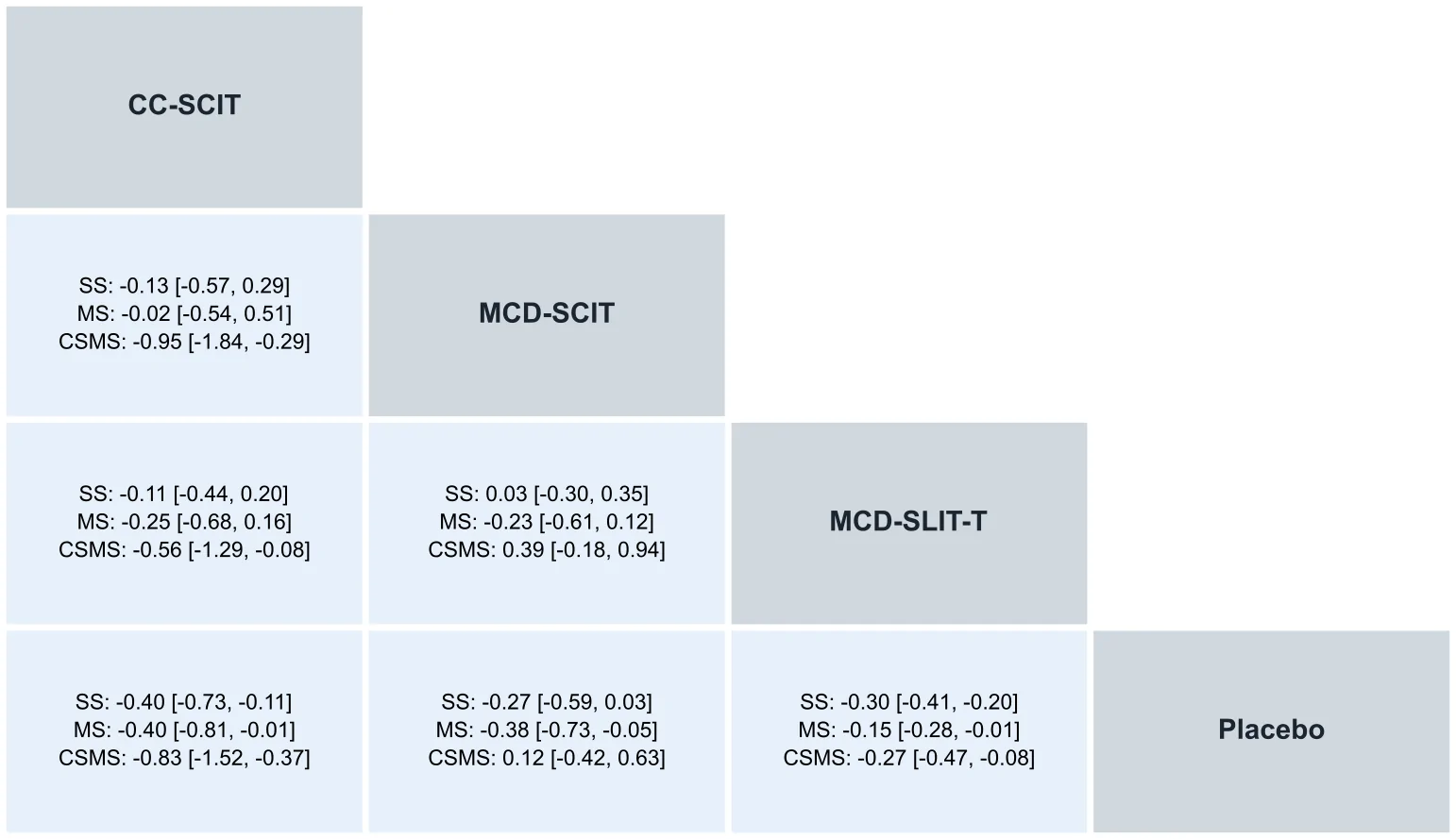
Network league table of indirect comparisons. League table showing standardized mean differences with 95% credible intervals for pairwise comparisons among treatment nodes derived from Bayesian network meta-analysis.

The network league table is shown in **Figure 5**. For symptom score and medication score, indirect comparisons among non-placebo treatment nodes were imprecise and the 95% CrIs crossed zero. For combined symptom and medication score, indirect estimates favored CC-SCIT over MCD-SLIT-tablet (SMD, −0.56; 95% CrI, −1.29 to −0.08) and over MCD-SCIT (SMD, −0.95; 95% CrI, −1.84 to −0.29). These comparisons should be interpreted as exploratory because no direct head-to-head comparisons among non-placebo interventions were available.

Comparison-adjusted funnel plots showed asymmetry for symptom score (Egger’s test, P = 0.039), but not for medication score (P = 0.793) or combined symptom and medication score (P = 0.134) (**Supplementary Figures 4A–C**). These findings were considered exploratory because each SCIT-versus-placebo comparison included no more than four trials and the amount of evidence was markedly imbalanced across treatment nodes. The observed symptom-score asymmetry could therefore reflect publication-related small-study effects, differences in sample size, trial era, or clinical characteristics across nodes, or a combination of these factors, and was not considered diagnostic of reporting bias. Leave-one-out analyses suggested that MCD-SLIT-tablet estimates for symptom score were stable, whereas SCIT estimates were more sensitive to individual studies. For CC-SCIT, omission of Bozek 2017 attenuated the symptom-score estimate to −0.32 (95% CrI, −0.69 to 0.01). For MCD-SCIT, omission of Suratannon 2024 strengthened the estimate to −0.47 (95% CrI, −0.87 to −0.07), whereas omission of Varney 2003 attenuated it to −0.13 (95% CrI, −0.45 to 0.19). Medication-score estimates were closest to the null and showed the greatest instability, particularly for SCIT nodes. For CSMS, CC-SCIT remained directionally favorable, but the effect varied from −2.19 after omitting Bachert 2025 to −0.32 after omitting Bozek 2017 (**Supplementary Figures 5A–C**).

Sensitivity analyses restricted to post-2005 trials did not materially alter symptom-score estimates for CC-SCIT (SMD, −0.39; 95% CrI, −0.70 to −0.11) or MCD-SLIT-tablet (SMD, −0.29; 95% CrI, −0.40 to −0.20), whereas the MCD-SCIT estimate was attenuated (SMD, −0.03; 95% CrI, −0.41 to 0.35). After excluding Japanese trials with near-zero baseline medication scores, medication-score estimates were −0.42 for CC-SCIT (95% CrI, −1.00 to 0.12), −0.41 for MCD-SCIT (95% CrI, −0.86 to −0.01), and −0.18 for MCD-SLIT-tablet (95% CrI, −0.47 to 0.11) (**Supplementary Figures 6**, **S7**). Prior-sensitivity analyses showed negligible differences between the uniform and half-normal heterogeneity prior specifications; all absolute differences in posterior mean SMDs were <0.01 (**Supplementary Table 3** in the **Supplementary Material**).

## 4 Discussion

In this network meta-analysis, we applied a predefined component-resolved framework to classify HDM-AIT interventions according to treatment-induced component-specific antibody profiles and product-level molecular characteristics, rather than administration route alone. CC-SCIT achieved the highest model-based rankings for symptom score and combined symptom and medication score, whereas MCD-SLIT-tablet provided the largest and most consistent placebo-controlled evidence base. These findings identify preparation-level allergen coverage as a clinically relevant treatment-defining characteristic and demonstrate the value of incorporating component-specific immunogenicity into evidence synthesis for HDM-AIT.

Previous NMAs have generally compared HDM-AIT according to administration route, most commonly SCIT versus SLIT (21, 55, 56). Although clinically intuitive, this approach may obscure clinically relevant differences among products. HDM-AIT preparations differ in source materials, manufacturing processes, standardization systems, and molecular allergen coverage. Our analysis complements previous evidence syntheses by applying a product-oriented, component-resolved framework, consistent with the regulatory and clinical principle that efficacy evidence generated for one AIT preparation should not automatically be extrapolated to another (10–13).

The comparison between CC-SCIT and MCD-SLIT-tablet reflects differences in both administration route and reported component profile and therefore does not isolate compositional breadth. The within-route comparison between CC-SCIT and MCD-SCIT is more informative regarding compositional differences, but it remains indirect and is supported by only three and four relatively small trials.

Within the MCD-SLIT-tablet node, IR and SQ-HDM represent manufacturer-specific potency systems and cannot be converted to a common dose scale. Although 6 and 12 SQ-HDM are expressed within the same standardization system, the included evidence largely reflects different regional regulatory programs and study populations. The node was therefore defined by the shared sublingual tablet route and major-component-dominant immunologic profile rather than by numerical equivalence of potency units. A formal dose-stratified analysis would have separated the evidence simultaneously by product, standardization system, regulatory region, age, and study population, while leaving only two to four trials in each subgroup. Such an analysis would not isolate a dose effect and would provide substantially less precise estimates. A previous tablet-specific meta-analysis likewise found no significant subgroup differences in combined symptom and medication score or rhinitis symptom score among the evaluated SQ-HDM and IR regimens (57).

The biological rationale for this framework is supported by component-resolved studies showing that HDM allergy is not limited to sensitization against major allergens such as Der p 1, Der p 2, and Der p 23. Many patients, particularly those with broader or more complex sensitization profiles, may also show IgE responses to intermediate or minor allergens, including Der p 4, Der p 5, Der p 7, and Der p 21 (16–18). In this context, the ability of an AIT preparation to induce blocking antibody responses beyond the major components becomes clinically relevant. Published immunologic profiling studies provide useful descriptive information on whether AIT preparations induce antibody responses predominantly to major allergens or more broadly across additional HDM components. Rodríguez-Domínguez et al. reported limited IgG4 responses to Der p 5, Der p 7, and Der p 21 with a major-component-dominant treatment pattern, whereas the COVER study reported broader IgG4 induction to intermediate HDM components, including Der p 5, Der p 7, and Der p 21, during treatment with a comprehensive-component preparation (16, 34). These studies do not prove that broader IgG4 induction necessarily mediates superior clinical efficacy, but they support the plausibility of using published component-level immunologic profiles as product-level descriptors in evidence synthesis.

Importantly, this does not imply that CC-SCIT is universally superior. Rather, it suggests that preparation-level allergen coverage may represent one of several factors potentially relevant to variability in treatment response. Patients with predominantly major allergen sensitization may still derive meaningful benefit from major-component-dominant preparations, as reflected by the consistent efficacy of MCD-SLIT-tablet versus placebo in our analysis. Conversely, whether patients with broader sensitization profiles derive differential benefit from preparations with expanded allergen representation remains an open question that requires prospective investigation. Because our analysis is based on aggregate trial-level data, it cannot directly assess patient-level treatment matching or sensitization-driven effect modification (19, 20).

The precision-medicine implication is therefore a testable matching hypothesis rather than an immediate treatment-selection rule. A patient sensitized almost exclusively to Der p 1 and Der p 2 may not require the same molecular coverage as a patient with clinically relevant recognition of Der p 5, Der p 7, Der p 21, or other components. Demonstrating a true matching effect would require individual participant data linking baseline component-specific IgE, verified product composition, treatment-induced blocking-antibody responses, and clinical outcomes.

These findings also remain clinically relevant in settings where component-resolved diagnostics are not routinely available. In such settings, including many practices in the United States, selection of HDM-AIT may continue to rely on a compatible clinical history together with skin testing using licensed standardized mite extracts or whole-extract serum-specific IgE. The component-resolved framework proposed here is intended primarily to improve preparation characterization and interpretation of product-specific evidence; it does not make molecular testing a prerequisite for AIT. When available, component testing may refine patient phenotyping, but when it is not available, clinicians should use product-specific clinical evidence and should not assume that preparations standardized to overall or major-allergen potency have identical coverage of additional HDM components (4, 15).

The efficacy findings also distinguish between the magnitude of the observed treatment signal and the maturity of the supporting evidence. CC-SCIT generated the most favorable model-based estimates for symptom-related outcomes, whereas MCD-SLIT-tablet produced more precise estimates supported by multiple large contemporary multicenter trials. These findings are complementary: CC-SCIT represents the strongest efficacy signal within the component-resolved network, while MCD-SLIT-tablet currently provides the most reproducible evidence of benefit versus placebo. MCD-SCIT occupied an intermediate position, with favorable estimates for some outcomes but fewer contributing trials. Interpretation of treatment rankings should therefore consider effect magnitude, precision, and the amount of evidence supporting each node together.

Sensitivity analyses provided additional information on the stability of these findings. MCD-SLIT-tablet estimates remained relatively consistent across leave-one-out and alternative-model analyses. SCIT estimates were more responsive to the omission of individual trials, reflecting the smaller number of available studies within these nodes. For CC-SCIT, the direction of benefit for combined symptom and medication score was maintained across sensitivity scenarios, although its magnitude varied. Medication-score estimates showed greater variability than symptom-score estimates, particularly among SCIT nodes. Collectively, these analyses indicate that the component-resolved efficacy pattern is informative while also identifying the treatment nodes for which additional randomized evidence would have the greatest value.

This study also establishes a methodological framework for component-resolved evidence synthesis in AIT. Public reporting of commercial extract composition remains inconsistent, and quantitative proteomic characterization is not available for every preparation. We therefore used a predefined hierarchy in which treatment-induced component-specific IgG4 response breadth served as the primary criterion for node assignment, with component-specific IgG and proteomic or product-level evidence providing additional support. This approach made the evidence underlying each node assignment explicit and reproducible. Wider adoption of standardized component-specific immunogenicity reporting, quantitative molecular characterization, and batch-level quality assessment would further strengthen product-oriented comparisons in future AIT trials.

Several design features strengthen the analysis. Eligibility was restricted to double-blind, placebo-controlled randomized trials with at least 12 months of treatment; the review was prospectively registered; direct pairwise estimates were presented alongside Bayesian network estimates; fixed-effect and random-effects model fit was compared; and conclusions were examined across leave-one-out, trial-era, floor-effect, and prior-distribution sensitivity analyses. In addition, the component-resolved framework made the assumptions underlying treatment-node assignment visible rather than leaving product heterogeneity implicit within broad route categories.

Several limitations should be acknowledged. First, the network was star-shaped, with no direct head-to-head comparisons among treatments, limiting inference on relative efficacy. Second, comparisons between CC-SCIT and MCD-SLIT-tablet reflect differences in both administration route and reported component profile; the contribution of compositional breadth therefore cannot be separated from that of administration route. Third, the SCIT nodes were supported by a small number of trials, increasing sensitivity to individual studies. Fourth, residual product-, standardization-, regional-, and population-level heterogeneity remained within the MCD-SLIT-tablet node, and clinical and methodological heterogeneity across trials may affect the transitivity assumption. Fifth, the analysis used aggregate data and cannot evaluate individual sensitization profiles or patient-level effect modification. Sixth, the assessment of small-study effects was insufficiently informative for the SCIT comparisons because each included no more than four trials; the funnel-plot findings should therefore be interpreted as exploratory. Finally, this analysis focused on efficacy outcomes and did not evaluate safety, adherence, or long-term disease modification.

In conclusion, this exploratory network meta-analysis identified an association between reported immunologic breadth of HDM-AIT preparation groups and variation in efficacy estimates across randomized trials. CC-SCIT generated the most favorable model-based signal for symptom-related outcomes, while MCD-SLIT-tablet provided the most extensive and stable evidence versus placebo. Together, these findings support treatment-induced component-specific IgG4 breadth and product-level molecular coverage as relevant dimensions of AIT evaluation. They also provide a rationale for future head-to-head and biomarker-stratified trials integrating verified preparation profiles, patient sensitization patterns, treatment-induced immune responses, and clinical outcomes.

## Data Availability

The original contributions presented in the study are included in the article/**Supplementary Material**. Further inquiries can be directed to the corresponding author.

## Supplementary material

Supplementary Figure 1 Domain-specific risk-of-bias assessment. Traffic light plot showing risk-of-bias judgments for each included randomized controlled trial using the revised Cochrane RoB 2 tool.

Supplementary Figure 2 Summary risk-of-bias assessment. Summary plot showing the proportion of included trials rated as low risk or some concerns across RoB 2 domains.

Supplementary Figure 3 Pairwise meta-analyses of clinical outcomes. Frequentist random-effects forest plots comparing each treatment node with placebo for (A) symptom score, (B) medication score, and (C) combined symptom and medication score. Effects are presented as standardized mean differences with 95% confidence intervals.

Supplementary Figure 4 Comparison-adjusted funnel plots for clinical outcomes. Comparison-adjusted funnel plots assessing small-study effects for (A) symptom score, (B) medication score, and (C) combined symptom and medication score. Because each SCIT-versus-placebo comparison included no more than four trials, these findings should be interpreted as exploratory.

Supplementary Figure 5 Leave-one-out sensitivity analyses for clinical outcomes. Leave-one-out sensitivity analyses evaluating the influence of individual trials on the Bayesian network estimates for (A) symptom score, (B) medication score, and (C) combined symptom and medication score.

Supplementary Figure 6 Sensitivity analysis excluding early trials. Bayesian network sensitivity analysis excluding trials published before 2005.

Supplementary Figure 7 Sensitivity analysis excluding near-zero baseline medication scores. Bayesian network sensitivity analysis excluding Japanese trials with near-zero baseline medication scores.

Supplementary Table 1 Full search strategies across all queried electronic databases.

Supplementary Table 2 Model fit and effect size comparisons between random-effects and fixed-effects models.

Supplementary Table 3 Sensitivity analysis using alternative prior distributions.

Supplementary Table 4 SUCRA values and posterior ranking uncertainty for each treatment and outcome.
