# Supplementary material for: Efficacy of house dust mite allergen immunotherapy in allergic rhinitis: a network meta-analysis based on component-resolved classification
Source: Front Immunol. 2026 Sep 3;17:1947642. doi: 10.3389/fimmu.2026.1947642 (PMC13582266; doi:10.3389/fimmu.2026.1947642)
Supplement: Supplementary file 1 [file DataSheet1.pdf]

Risk of bias domains

Study	D1	D2	D3	D4	D5	Overall
McHugh 1990	-	+	+	+	-	-
Varney 2003	+	+	+	+	-	-
Xian 2019	+	+	+	+	+	+
Suratannon 2024	+	+	+	+	+	+
Yukselen 2012	+	+	+	+	-	-
Bozek 2017	+	+	+	+	+	+
Bachert 2025	+	+	+	+	-	-
Bergmann 2014	+	+	+	+	+	+
Demoly 2016	+	+	-	+	+	-
Nolte 2016	+	+	+	+	+	+
Okamoto 2017	+	+	+	+	+	+
Okubo 2017	+	+	+	+	+	+
Masuyama 2018	+	+	+	+	+	+
Okamoto 2019	+	+	+	+	+	+
Demoly 2020	+	+	-	+	+	-
Schuster 2025	+	+	+	+	+	+

Domains:
D1: Bias due to randomisation.
D2: Bias due to deviations from intended intervention.
D3: Bias due to missing data.
D4: Bias due to outcome measurement.
D5: Bias due to selection of reported result.

Judgement
+ Low
- Some concerns
