# Supplementary material for: Efficacy of house dust mite allergen immunotherapy in allergic rhinitis: a network meta-analysis based on component-resolved classification
Source: Front Immunol. 2026 Sep 3;17:1947642. doi: 10.3389/fimmu.2026.1947642 (PMC13582266; doi:10.3389/fimmu.2026.1947642)
Supplement: Supplementary file 3 [file DataSheet3.pdf]

(A)

Study (SS)	AIT Group Mean±SD (N)	Placebo Group Mean±SD (N)	SMD (95% CI)
CC-SCIT			
Yukselen 2012	2.8±1.2 (10)	4.0±1.1 (10)	−1.01 (−1.96, −0.07)
Bozek 2017	1.3±0.6 (29)	1.9±1.1 (26)	−0.66 (−1.21, −0.12)
Bachert 2025	0.8±0.7 (105)	1.0±0.7 (123)	−0.23 (−0.49, 0.03)
**Random effects model**	**Total N=144**	**Total N=159**	**−0.49 (−0.92, −0.06)**
Heterogeneity: I² = 50%, τ² = 0.072, p = 0.137			
MCD-SCIT			
McHugh 1990	16.0±14.3 (27)	22.4±18.3 (28)	−0.38 (−0.92, 0.15)
Varney 2003	40.0±46.5 (15)	111.0±64.9 (13)	−1.24 (−2.06, −0.42)
Xian 2019	1.8±1.8 (26)	2.0±1.3 (14)	−0.08 (−0.73, 0.57)
Suratannon 2024	4.1±1.6 (96)	4.1±3.6 (34)	0.00 (−0.39, 0.39)
**Random effects model**	**Total N=164**	**Total N=89**	**−0.35 (−0.82, 0.12)**
Heterogeneity: I² = 63%, τ² = 0.139, p = 0.055			
MCD-SLIT-tablet			
Bergmann 2014	2.7±1.1 (141)	3.3±1.1 (153)	−0.57 (−0.80, −0.33)
Demoly 2016	2.8±2.2 (284)	3.3±2.2 (298)	−0.25 (−0.41, −0.08)
Nolte 2016	3.5±2.5 (566)	4.2±3.0 (620)	−0.23 (−0.35, −0.12)
Okamoto 2017	5.0±3.7 (315)	6.0±3.7 (316)	−0.29 (−0.44, −0.13)
Okubo 2017	3.7±2.7 (285)	4.8±2.7 (285)	−0.39 (−0.56, −0.23)
Masuyama 2018	4.0±2.6 (209)	5.1±2.6 (218)	−0.44 (−0.64, −0.25)
Okamoto 2019	6.2±2.7 (205)	7.2±2.8 (217)	−0.33 (−0.52, −0.14)
Demoly 2020	2.9±3.2 (586)	3.6±3.2 (676)	−0.21 (−0.32, −0.10)
Schuster 2025	1.5±2.6 (693)	1.9±2.7 (706)	−0.15 (−0.26, −0.05)
**Random effects model**	**Total N=3284**	**Total N=3489**	**−0.29 (−0.37, −0.22)**
Heterogeneity: I² = 58%, τ² = 0.008, p = 0.018			
**Overall Random effects model**	**Total N=3592**	**Total N=3737**	**−0.30 (−0.37, −0.23)**
Overall Heterogeneity: I² = 45%, τ² = 0.008, p = 0.010			

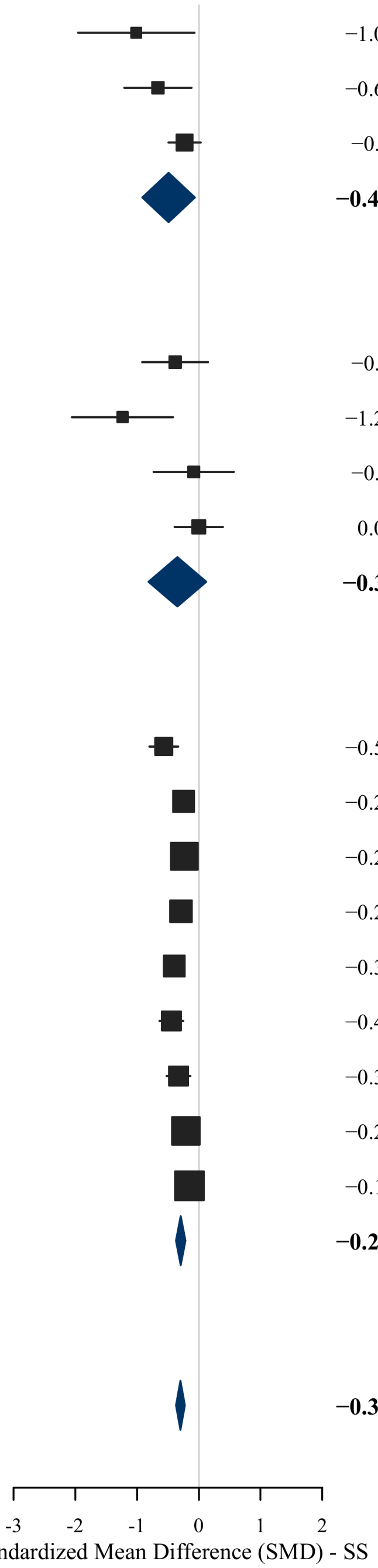

(B)

Study (MS)	AIT Group Mean±SD (N)	Placebo Group Mean±SD (N)	SMD (95% CI)
CC-SCIT			
Yukselen 2012	1.0±1.5 (10)	2.0±1.2 (10)	−0.67 (−1.58, 0.23)
Bachert 2025	0.4±0.7 (105)	0.6±0.7 (123)	−0.34 (−0.61, −0.08)
**Random effects model**	**Total N=115**	**Total N=133**	**−0.37 (−0.62, −0.12)**
Heterogeneity: $I^2 = 0\%$, $\tau^2 = 0.000$, $p = 0.494$			
MCD-SCIT			
McHugh 1990	0.2±0.6 (27)	1.0±2.0 (28)	−0.51 (−1.05, 0.03)
Varney 2003	18.0±13.9 (15)	32.0±50.5 (13)	−0.38 (−1.13, 0.37)
Xian 2020	1.6±2.5 (26)	4.8±2.4 (14)	−1.23 (−1.94, −0.53)
Suratannon 2024	1.2±1.5 (96)	1.1±1.0 (34)	0.07 (−0.32, 0.46)
**Random effects model**	**Total N=164**	**Total N=89**	**−0.47 (−1.00, 0.07)**
Heterogeneity: $I^2 = 71\%$, $\tau^2 = 0.209$, $p = 0.013$			
MCD-SLIT-tablet			
Bergmann 2014	0.3±0.3 (141)	0.3±0.3 (153)	0.03 (−0.20, 0.26)
Demoly 2016	2.2±3.2 (284)	2.8±3.2 (298)	−0.19 (−0.35, −0.03)
Nolte 2016	0.6±0.3 (566)	0.8±0.3 (620)	−0.44 (−0.55, −0.32)
Okamoto 2017	0.0±0.4 (315)	0.1±0.4 (316)	−0.08 (−0.24, 0.07)
Okubo 2017	0.1±0.4 (285)	0.1±0.4 (285)	−0.11 (−0.28, 0.05)
Masuyama 2018	0.0±0.2 (209)	0.1±0.2 (218)	−0.19 (−0.38, 0.00)
Okamoto 2019	0.1±0.2 (205)	0.1±0.2 (217)	0.00 (−0.19, 0.19)
Demoly 2020	0.2±0.4 (586)	0.3±0.4 (676)	−0.20 (−0.31, −0.09)
Schuster 2025	1.4±5.3 (693)	1.9±5.3 (706)	−0.09 (−0.20, 0.01)
**Random effects model**	**Total N=3284**	**Total N=3489**	**−0.15 (−0.25, −0.06)**
Heterogeneity: $I^2 = 72\%$, $\tau^2 = 0.014$, $p < 0.001$			
**Overall Random effects model**	**Total N=3563**	**Total N=3711**	**−0.19 (−0.28, −0.10)**
Overall Heterogeneity: $I^2 = 65\%$, $\tau^2 = 0.016$, $p < 0.001$			

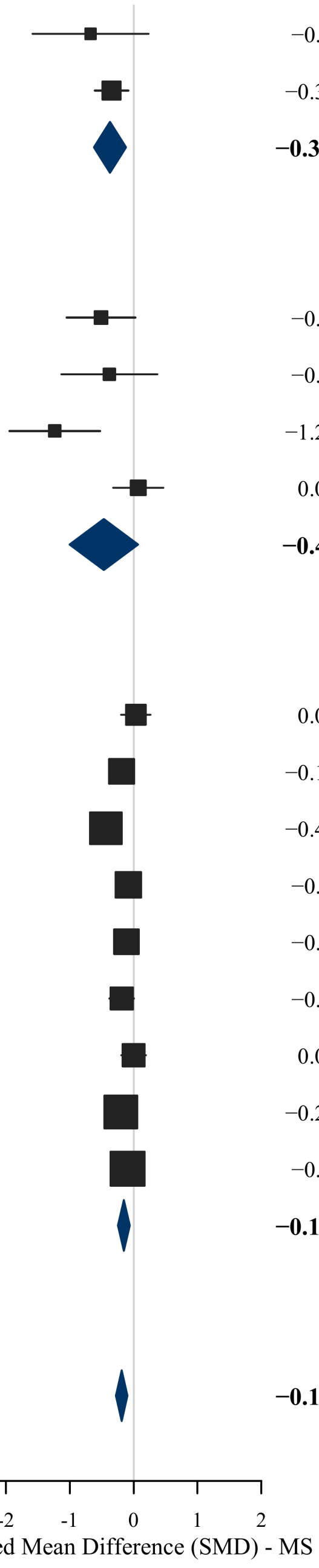

(C)

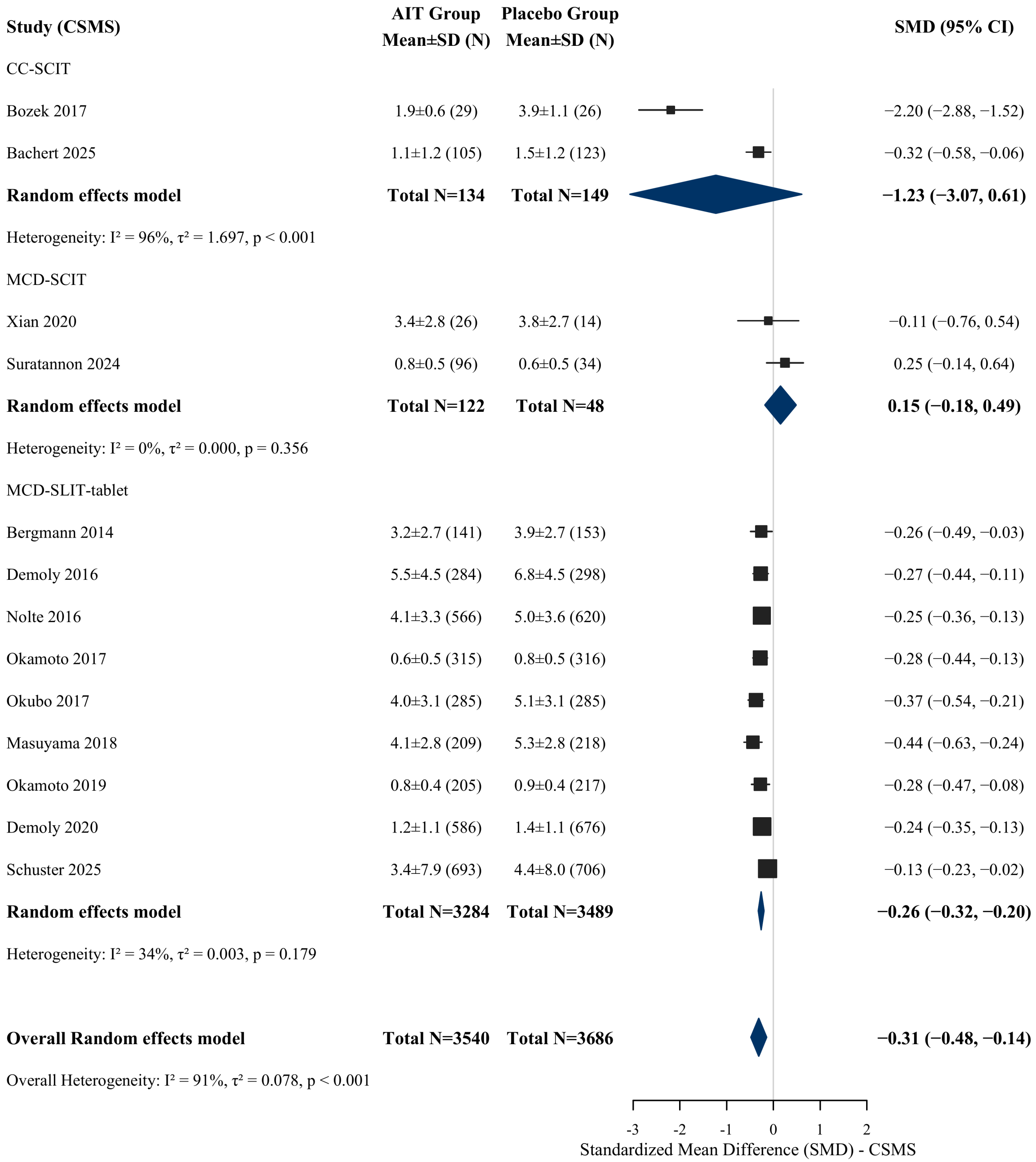

Study (CSMS)
AIT Group Mean±SD (N)
Placebo Group Mean±SD (N)
SMD (95% CI)
CC-SCIT
Bozek 2017 | 1.9±0.6 (29) | 3.9±1.1 (26) | −2.20 (−2.88, −1.52)
Bachert 2025 | 1.1±1.2 (105) | 1.5±1.2 (123) | −0.32 (−0.58, −0.06)
Random effects model | Total N=134 | Total N=149 | −1.23 (−3.07, 0.61)
Heterogeneity: I² = 96%, τ² = 1.697, p < 0.001
MCD-SCIT
Xian 2020 | 3.4±2.8 (26) | 3.8±2.7 (14) | −0.11 (−0.76, 0.54)
Suratannon 2024 | 0.8±0.5 (96) | 0.6±0.5 (34) | 0.25 (−0.14, 0.64)
Random effects model | Total N=122 | Total N=48 | 0.15 (−0.18, 0.49)
Heterogeneity: I² = 0%, τ² = 0.000, p = 0.356
MCD-SLIT-tablet
Bergmann 2014 | 3.2±2.7 (141) | 3.9±2.7 (153) | −0.26 (−0.49, −0.03)
Demoly 2016 | 5.5±4.5 (284) | 6.8±4.5 (298) | −0.27 (−0.44, −0.11)
Nolte 2016 | 4.1±3.3 (566) | 5.0±3.6 (620) | −0.25 (−0.36, −0.13)
Okamoto 2017 | 0.6±0.5 (315) | 0.8±0.5 (316) | −0.28 (−0.44, −0.13)
Okubo 2017 | 4.0±3.1 (285) | 5.1±3.1 (285) | −0.37 (−0.54, −0.21)
Masuyama 2018 | 4.1±2.8 (209) | 5.3±2.8 (218) | −0.44 (−0.63, −0.24)
Okamoto 2019 | 0.8±0.4 (205) | 0.9±0.4 (217) | −0.28 (−0.47, −0.08)
Demoly 2020 | 1.2±1.1 (586) | 1.4±1.1 (676) | −0.24 (−0.35, −0.13)
Schuster 2025 | 3.4±7.9 (693) | 4.4±8.0 (706) | −0.13 (−0.23, −0.02)
Random effects model | Total N=3284 | Total N=3489 | −0.26 (−0.32, −0.20)
Heterogeneity: I² = 34%, τ² = 0.003, p = 0.179
Overall Random effects model | Total N=3540 | Total N=3686 | −0.31 (−0.48, −0.14)
Overall Heterogeneity: I² = 91%, τ² = 0.078, p < 0.001
-3
-2
-1
0
1
2
Standardized Mean Difference (SMD) - CSMS
