# Supplementary material for: Efficacy of house dust mite allergen immunotherapy in allergic rhinitis: a network meta-analysis based on component-resolved classification
Source: Front Immunol. 2026 Sep 3;17:1947642. doi: 10.3389/fimmu.2026.1947642 (PMC13582266; doi:10.3389/fimmu.2026.1947642)
Supplement: Supplementary file 4 [file DataSheet4.pdf]

(A)

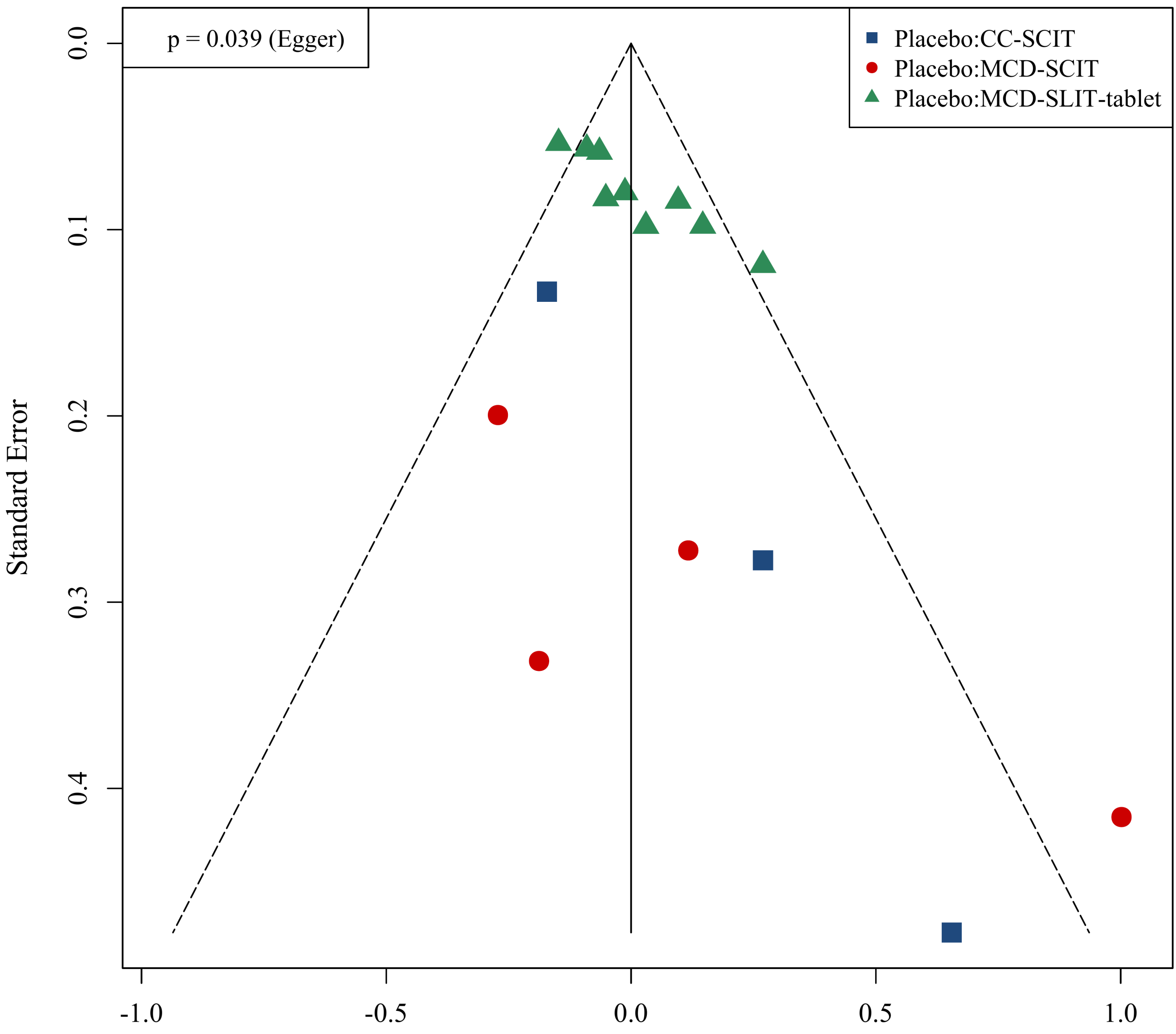
p = 0.039 (Egger)
Placebo:CC-SCIT
Placebo:MCD-SCIT
Placebo:MCD-SLIT-tablet
Standard Error
0.0
0.1
0.2
0.3
0.4
-1.0
-0.5
0.0
0.5
1.0

(B)

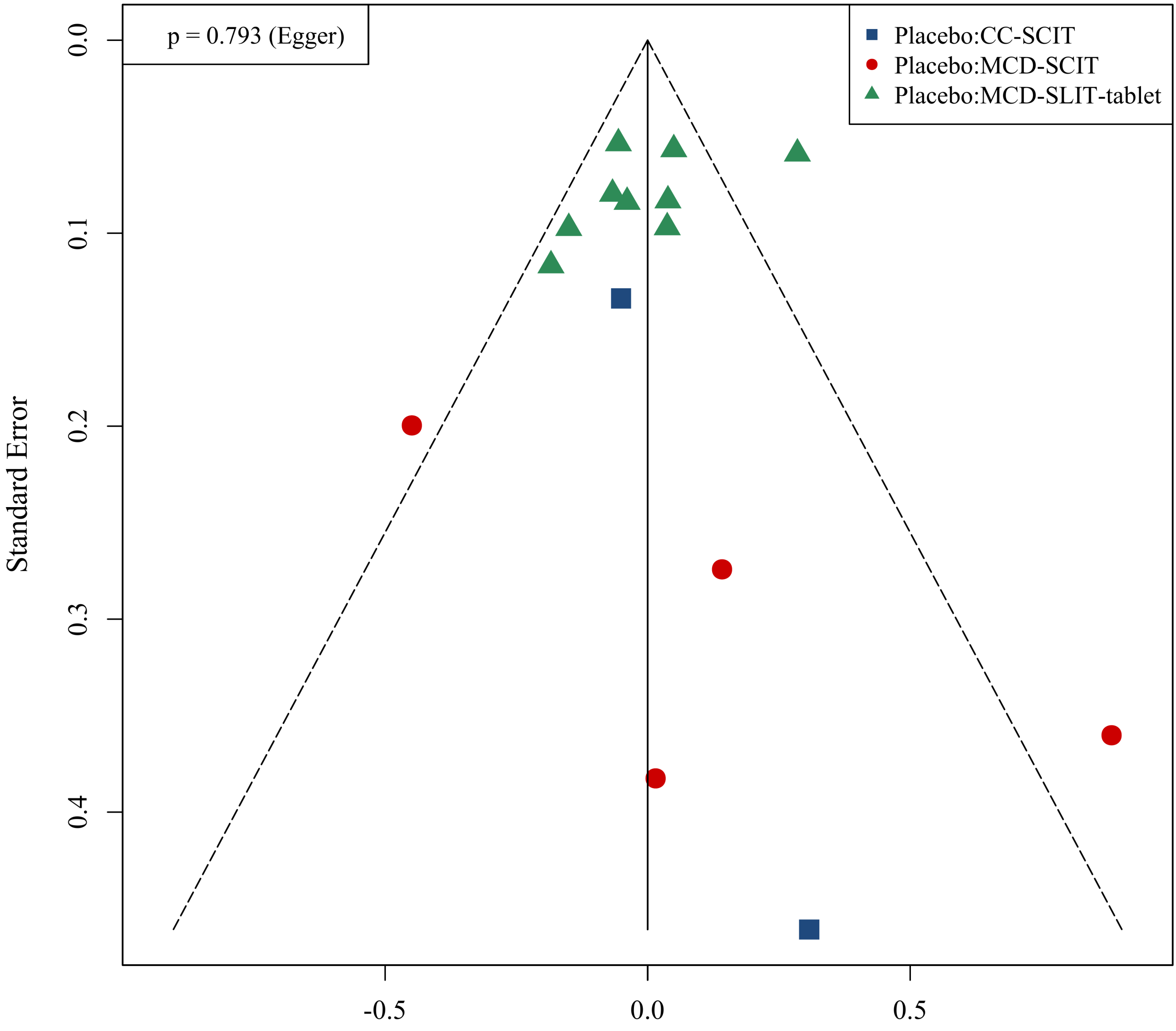

p = 0.793 (Egger)
Placebo:CC-SCIT
Placebo:MCD-SCIT
Placebo:MCD-SLIT-tablet
0.0
0.1
0.2
0.3
0.4
Standard Error
-0.5
0.0
0.5

(C)

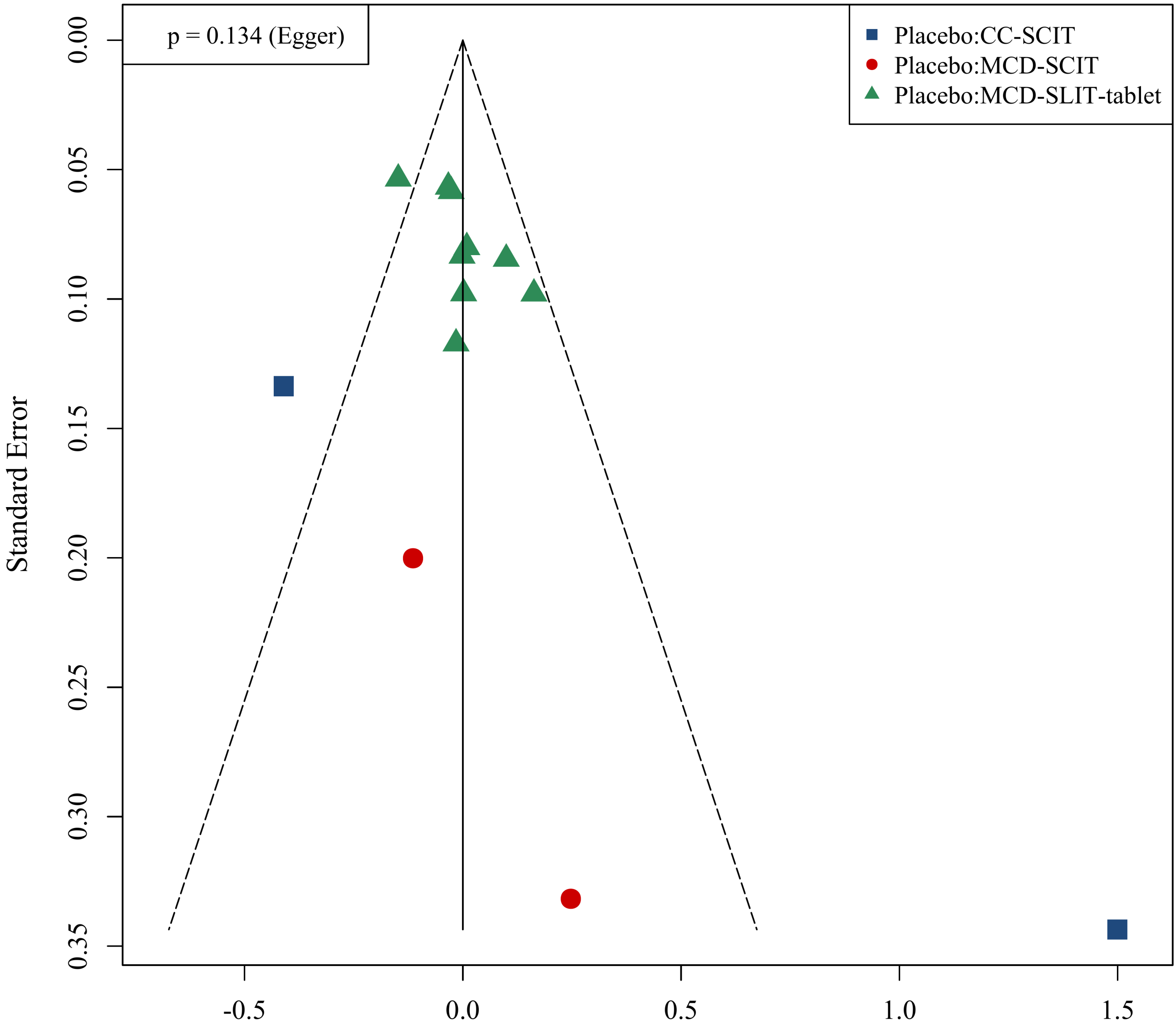
p = 0.134 (Egger)
Placebo:CC-SCIT
Placebo:MCD-SCIT
Placebo:MCD-SLIT-tablet
Standard Error
0.00
0.05
0.10
0.15
0.20
0.25
0.30
0.35
-0.5
0.0
0.5
1.0
1.5
