# Supplementary material for: Efficacy of house dust mite allergen immunotherapy in allergic rhinitis: a network meta-analysis based on component-resolved classification
Source: Front Immunol. 2026 Sep 3;17:1947642. doi: 10.3389/fimmu.2026.1947642 (PMC13582266; doi:10.3389/fimmu.2026.1947642)
Supplement: Supplementary file 5 [file DataSheet5.pdf]

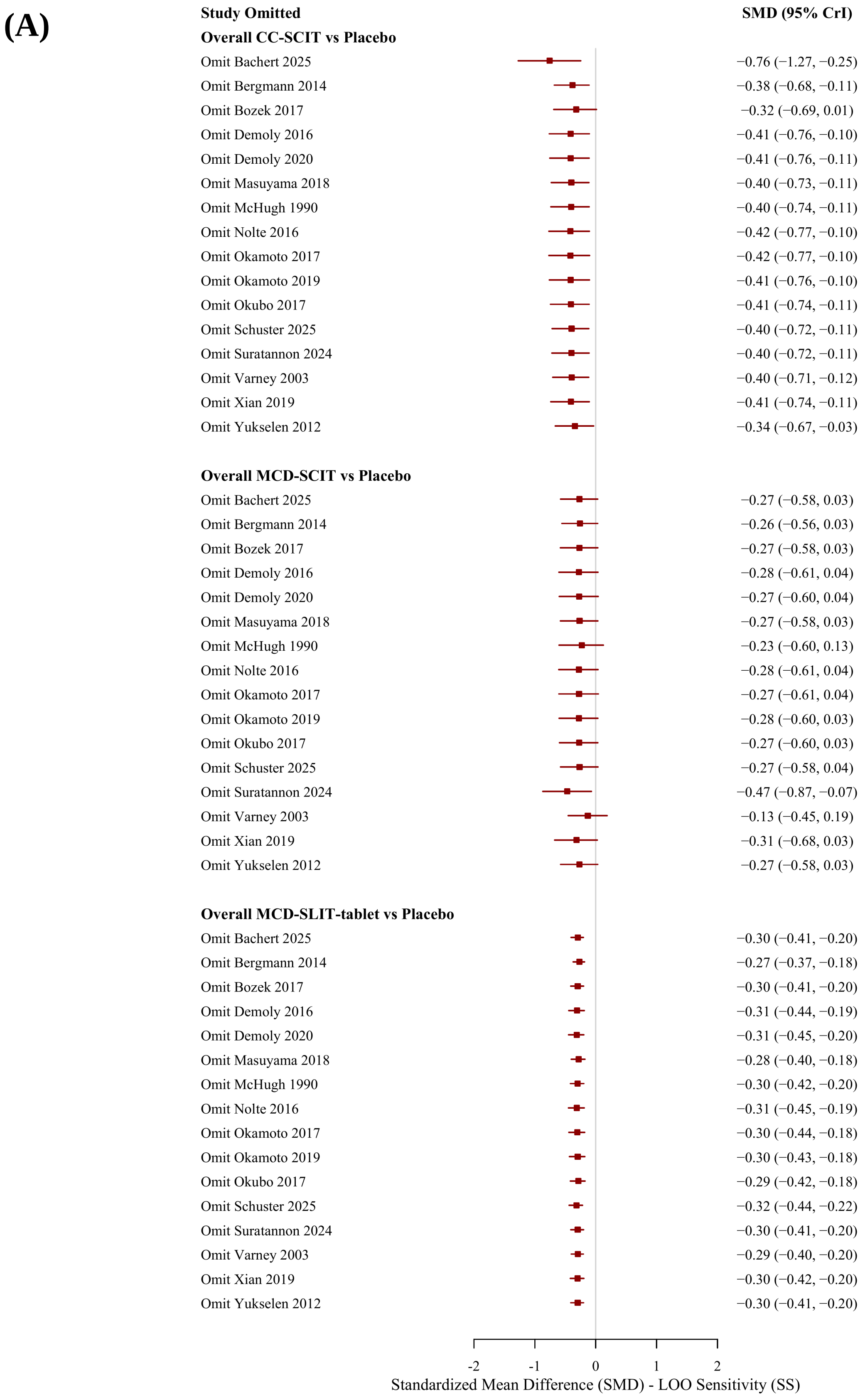
(A)
Study Omitted
SMD (95% CrI)
Overall CC-SCIT vs Placebo
Omit Bachert 2025 −0.76 (−1.27, −0.25)
Omit Bergmann 2014 −0.38 (−0.68, −0.11)
Omit Bozek 2017 −0.32 (−0.69, 0.01)
Omit Demoly 2016 −0.41 (−0.76, −0.10)
Omit Demoly 2020 −0.41 (−0.76, −0.11)
Omit Masuyama 2018 −0.40 (−0.73, −0.11)
Omit McHugh 1990 −0.40 (−0.74, −0.11)
Omit Nolte 2016 −0.42 (−0.77, −0.10)
Omit Okamoto 2017 −0.42 (−0.77, −0.10)
Omit Okamoto 2019 −0.41 (−0.76, −0.10)
Omit Okubo 2017 −0.41 (−0.74, −0.11)
Omit Schuster 2025 −0.40 (−0.72, −0.11)
Omit Suratannon 2024 −0.40 (−0.72, −0.11)
Omit Varney 2003 −0.40 (−0.71, −0.12)
Omit Xian 2019 −0.41 (−0.74, −0.11)
Omit Yukselen 2012 −0.34 (−0.67, −0.03)
Overall MCD-SCIT vs Placebo
Omit Bachert 2025 −0.27 (−0.58, 0.03)
Omit Bergmann 2014 −0.26 (−0.56, 0.03)
Omit Bozek 2017 −0.27 (−0.58, 0.03)
Omit Demoly 2016 −0.28 (−0.61, 0.04)
Omit Demoly 2020 −0.27 (−0.60, 0.04)
Omit Masuyama 2018 −0.27 (−0.58, 0.03)
Omit McHugh 1990 −0.23 (−0.60, 0.13)
Omit Nolte 2016 −0.28 (−0.61, 0.04)
Omit Okamoto 2017 −0.27 (−0.61, 0.04)
Omit Okamoto 2019 −0.28 (−0.60, 0.03)
Omit Okubo 2017 −0.27 (−0.60, 0.03)
Omit Schuster 2025 −0.27 (−0.58, 0.04)
Omit Suratannon 2024 −0.47 (−0.87, −0.07)
Omit Varney 2003 −0.13 (−0.45, 0.19)
Omit Xian 2019 −0.31 (−0.68, 0.03)
Omit Yukselen 2012 −0.27 (−0.58, 0.03)
Overall MCD-SLIT-tablet vs Placebo
Omit Bachert 2025 −0.30 (−0.41, −0.20)
Omit Bergmann 2014 −0.27 (−0.37, −0.18)
Omit Bozek 2017 −0.30 (−0.41, −0.20)
Omit Demoly 2016 −0.31 (−0.44, −0.19)
Omit Demoly 2020 −0.31 (−0.45, −0.20)
Omit Masuyama 2018 −0.28 (−0.40, −0.18)
Omit McHugh 1990 −0.30 (−0.42, −0.20)
Omit Nolte 2016 −0.31 (−0.45, −0.19)
Omit Okamoto 2017 −0.30 (−0.44, −0.18)
Omit Okamoto 2019 −0.30 (−0.43, −0.18)
Omit Okubo 2017 −0.29 (−0.42, −0.18)
Omit Schuster 2025 −0.32 (−0.44, −0.22)
Omit Suratannon 2024 −0.30 (−0.41, −0.20)
Omit Varney 2003 −0.29 (−0.40, −0.20)
Omit Xian 2019 −0.30 (−0.42, −0.20)
Omit Yukselen 2012 −0.30 (−0.41, −0.20)
-2
-1
0
1
2
Standardized Mean Difference (SMD) - LOO Sensitivity (SS)

(B)

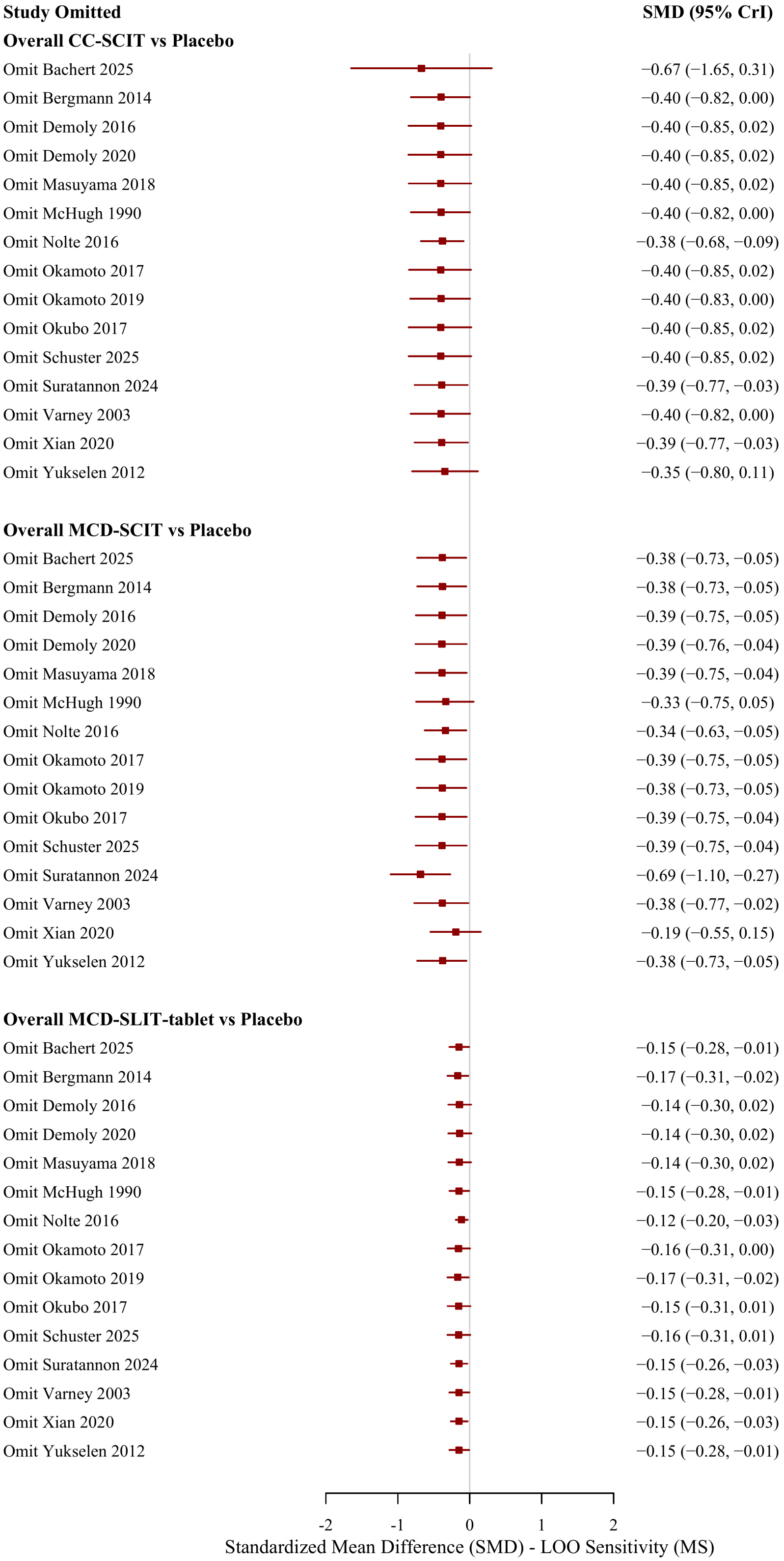

Study Omitted
SMD (95% CrI)
Overall CC-SCIT vs Placebo
Omit Bachert 2025 −0.67 (−1.65, 0.31)
Omit Bergmann 2014 −0.40 (−0.82, 0.00)
Omit Demoly 2016 −0.40 (−0.85, 0.02)
Omit Demoly 2020 −0.40 (−0.85, 0.02)
Omit Masuyama 2018 −0.40 (−0.85, 0.02)
Omit McHugh 1990 −0.40 (−0.82, 0.00)
Omit Nolte 2016 −0.38 (−0.68, −0.09)
Omit Okamoto 2017 −0.40 (−0.85, 0.02)
Omit Okamoto 2019 −0.40 (−0.83, 0.00)
Omit Okubo 2017 −0.40 (−0.85, 0.02)
Omit Schuster 2025 −0.40 (−0.85, 0.02)
Omit Suratannon 2024 −0.39 (−0.77, −0.03)
Omit Varney 2003 −0.40 (−0.82, 0.00)
Omit Xian 2020 −0.39 (−0.77, −0.03)
Omit Yukselen 2012 −0.35 (−0.80, 0.11)
Overall MCD-SCIT vs Placebo
Omit Bachert 2025 −0.38 (−0.73, −0.05)
Omit Bergmann 2014 −0.38 (−0.73, −0.05)
Omit Demoly 2016 −0.39 (−0.75, −0.05)
Omit Demoly 2020 −0.39 (−0.76, −0.04)
Omit Masuyama 2018 −0.39 (−0.75, −0.04)
Omit McHugh 1990 −0.33 (−0.75, 0.05)
Omit Nolte 2016 −0.34 (−0.63, −0.05)
Omit Okamoto 2017 −0.39 (−0.75, −0.05)
Omit Okamoto 2019 −0.38 (−0.73, −0.05)
Omit Okubo 2017 −0.39 (−0.75, −0.04)
Omit Schuster 2025 −0.39 (−0.75, −0.04)
Omit Suratannon 2024 −0.69 (−1.10, −0.27)
Omit Varney 2003 −0.38 (−0.77, −0.02)
Omit Xian 2020 −0.19 (−0.55, 0.15)
Omit Yukselen 2012 −0.38 (−0.73, −0.05)
Overall MCD-SLIT-tablet vs Placebo
Omit Bachert 2025 −0.15 (−0.28, −0.01)
Omit Bergmann 2014 −0.17 (−0.31, −0.02)
Omit Demoly 2016 −0.14 (−0.30, 0.02)
Omit Demoly 2020 −0.14 (−0.30, 0.02)
Omit Masuyama 2018 −0.14 (−0.30, 0.02)
Omit McHugh 1990 −0.15 (−0.28, −0.01)
Omit Nolte 2016 −0.12 (−0.20, −0.03)
Omit Okamoto 2017 −0.16 (−0.31, 0.00)
Omit Okamoto 2019 −0.17 (−0.31, −0.02)
Omit Okubo 2017 −0.15 (−0.31, 0.01)
Omit Schuster 2025 −0.16 (−0.31, 0.01)
Omit Suratannon 2024 −0.15 (−0.26, −0.03)
Omit Varney 2003 −0.15 (−0.28, −0.01)
Omit Xian 2020 −0.15 (−0.26, −0.03)
Omit Yukselen 2012 −0.15 (−0.28, −0.01)
-2
-1
0
1
2
Standardized Mean Difference (SMD) - LOO Sensitivity (MS)

(C)

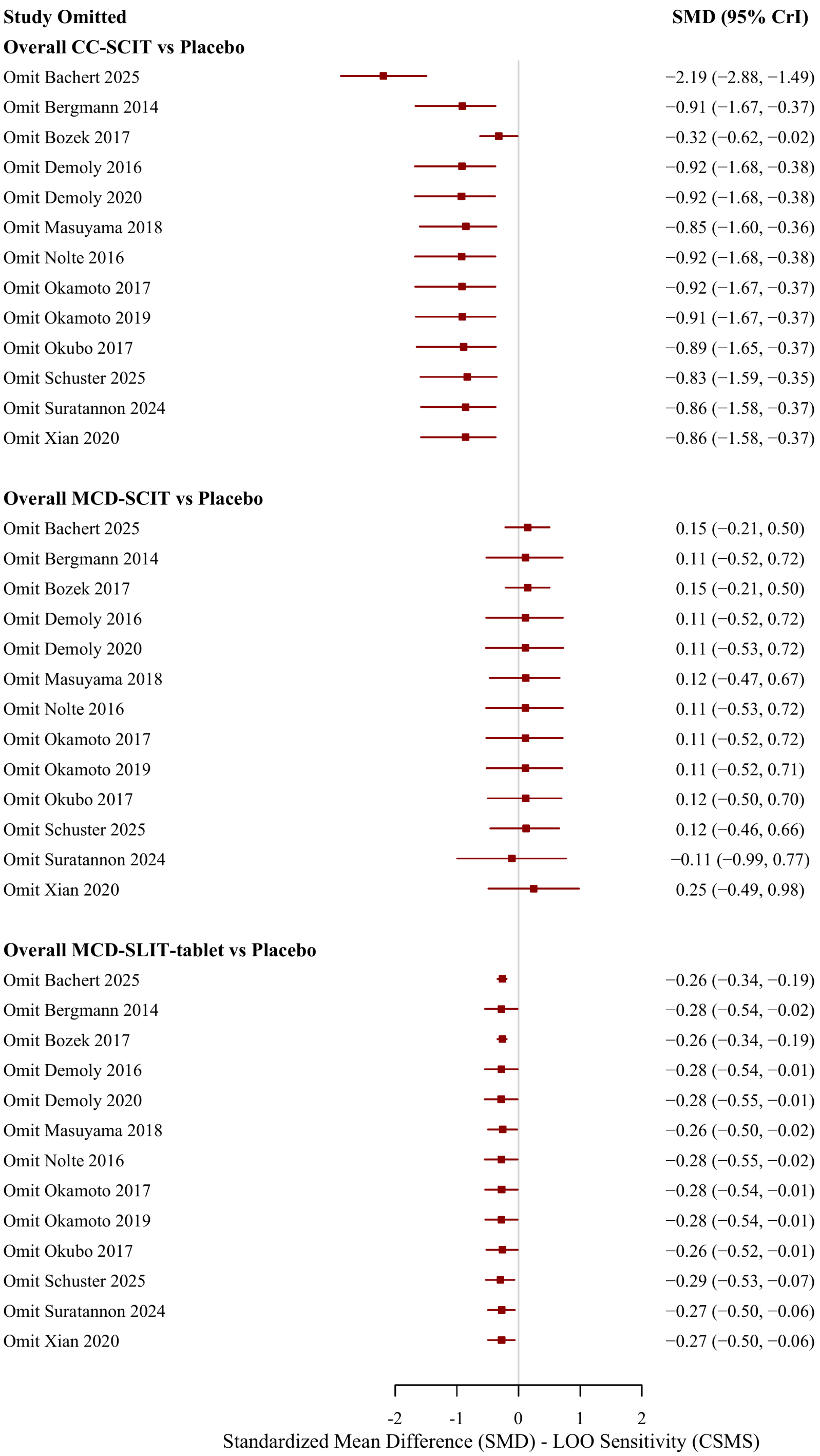

Study Omitted
SMD (95% CrI)
Overall CC-SCIT vs Placebo
Omit Bachert 2025 −2.19 (−2.88, −1.49)
Omit Bergmann 2014 −0.91 (−1.67, −0.37)
Omit Bozek 2017 −0.32 (−0.62, −0.02)
Omit Demoly 2016 −0.92 (−1.68, −0.38)
Omit Demoly 2020 −0.92 (−1.68, −0.38)
Omit Masuyama 2018 −0.85 (−1.60, −0.36)
Omit Nolte 2016 −0.92 (−1.68, −0.38)
Omit Okamoto 2017 −0.92 (−1.67, −0.37)
Omit Okamoto 2019 −0.91 (−1.67, −0.37)
Omit Okubo 2017 −0.89 (−1.65, −0.37)
Omit Schuster 2025 −0.83 (−1.59, −0.35)
Omit Suratannon 2024 −0.86 (−1.58, −0.37)
Omit Xian 2020 −0.86 (−1.58, −0.37)
Overall MCD-SCIT vs Placebo
Omit Bachert 2025 0.15 (−0.21, 0.50)
Omit Bergmann 2014 0.11 (−0.52, 0.72)
Omit Bozek 2017 0.15 (−0.21, 0.50)
Omit Demoly 2016 0.11 (−0.52, 0.72)
Omit Demoly 2020 0.11 (−0.53, 0.72)
Omit Masuyama 2018 0.12 (−0.47, 0.67)
Omit Nolte 2016 0.11 (−0.53, 0.72)
Omit Okamoto 2017 0.11 (−0.52, 0.72)
Omit Okamoto 2019 0.11 (−0.52, 0.71)
Omit Okubo 2017 0.12 (−0.50, 0.70)
Omit Schuster 2025 0.12 (−0.46, 0.66)
Omit Suratannon 2024 −0.11 (−0.99, 0.77)
Omit Xian 2020 0.25 (−0.49, 0.98)
Overall MCD-SLIT-tablet vs Placebo
Omit Bachert 2025 −0.26 (−0.34, −0.19)
Omit Bergmann 2014 −0.28 (−0.54, −0.02)
Omit Bozek 2017 −0.26 (−0.34, −0.19)
Omit Demoly 2016 −0.28 (−0.54, −0.01)
Omit Demoly 2020 −0.28 (−0.55, −0.01)
Omit Masuyama 2018 −0.26 (−0.50, −0.02)
Omit Nolte 2016 −0.28 (−0.55, −0.02)
Omit Okamoto 2017 −0.28 (−0.54, −0.01)
Omit Okamoto 2019 −0.28 (−0.54, −0.01)
Omit Okubo 2017 −0.26 (−0.52, −0.01)
Omit Schuster 2025 −0.29 (−0.53, −0.07)
Omit Suratannon 2024 −0.27 (−0.50, −0.06)
Omit Xian 2020 −0.27 (−0.50, −0.06)
-2
-1
0
1
2
Standardized Mean Difference (SMD) - LOO Sensitivity (CSMS)
