# Supplementary material for: Efficacy of house dust mite allergen immunotherapy in allergic rhinitis: a network meta-analysis based on component-resolved classification
Source: Front Immunol. 2026 Sep 3;17:1947642. doi: 10.3389/fimmu.2026.1947642 (PMC13582266; doi:10.3389/fimmu.2026.1947642)
Supplement: Supplementary file 6 [file DataSheet6.pdf]

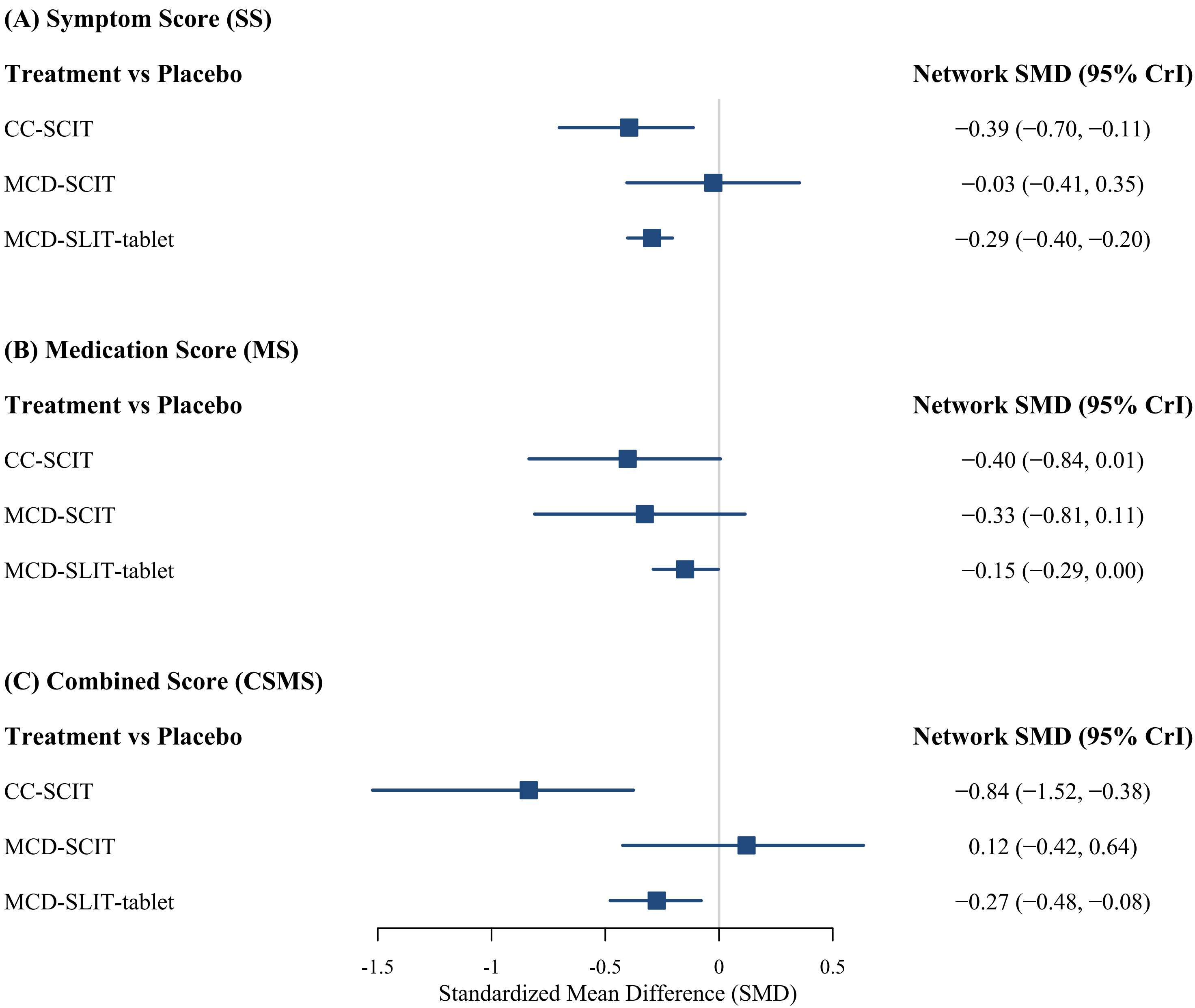
(A) Symptom Score (SS)
Treatment vs Placebo
Network SMD (95% CrI)
CC-SCIT
−0.39 (−0.70, −0.11)
MCD-SCIT
−0.03 (−0.41, 0.35)
MCD-SLIT-tablet
−0.29 (−0.40, −0.20)
(B) Medication Score (MS)
Treatment vs Placebo
Network SMD (95% CrI)
CC-SCIT
−0.40 (−0.84, 0.01)
MCD-SCIT
−0.33 (−0.81, 0.11)
MCD-SLIT-tablet
−0.15 (−0.29, 0.00)
(C) Combined Score (CSMS)
Treatment vs Placebo
Network SMD (95% CrI)
CC-SCIT
−0.84 (−1.52, −0.38)
MCD-SCIT
0.12 (−0.42, 0.64)
MCD-SLIT-tablet
−0.27 (−0.48, −0.08)
-1.5
-1
-0.5
0
0.5
Standardized Mean Difference (SMD)
