# Supplementary material for: Efficacy of house dust mite allergen immunotherapy in allergic rhinitis: a network meta-analysis based on component-resolved classification
Source: Front Immunol. 2026 Sep 3;17:1947642. doi: 10.3389/fimmu.2026.1947642 (PMC13582266; doi:10.3389/fimmu.2026.1947642)
Supplement: Supplementary file 7 [file DataSheet7.pdf]

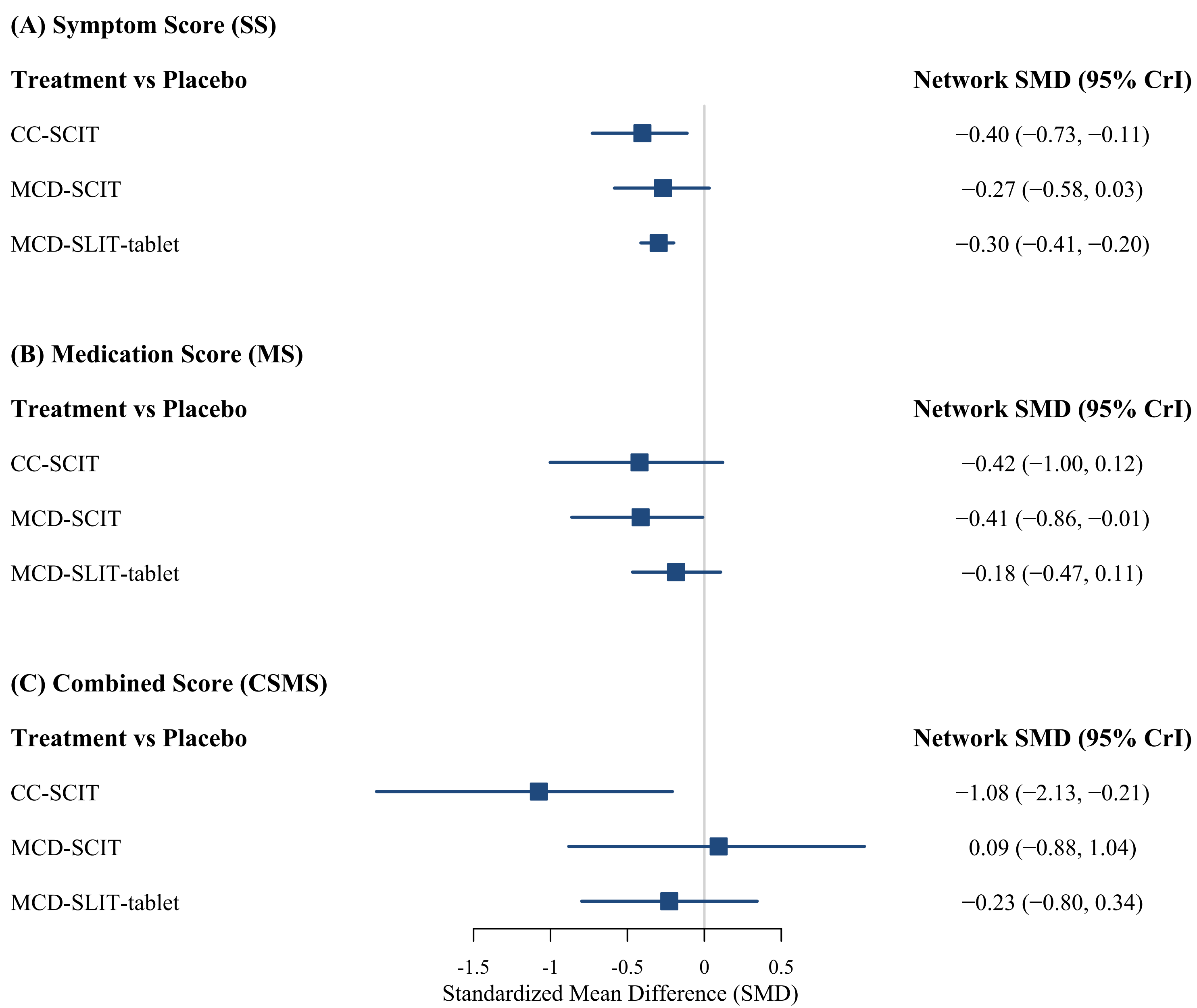
(A) Symptom Score (SS)
Treatment vs Placebo
Network SMD (95% CrI)
CC-SCIT
−0.40 (−0.73, −0.11)
MCD-SCIT
−0.27 (−0.58, 0.03)
MCD-SLIT-tablet
−0.30 (−0.41, −0.20)
(B) Medication Score (MS)
Treatment vs Placebo
Network SMD (95% CrI)
CC-SCIT
−0.42 (−1.00, 0.12)
MCD-SCIT
−0.41 (−0.86, −0.01)
MCD-SLIT-tablet
−0.18 (−0.47, 0.11)
(C) Combined Score (CSMS)
Treatment vs Placebo
Network SMD (95% CrI)
CC-SCIT
−1.08 (−2.13, −0.21)
MCD-SCIT
0.09 (−0.88, 1.04)
MCD-SLIT-tablet
−0.23 (−0.80, 0.34)
-1.5
-1
-0.5
0
0.5
Standardized Mean Difference (SMD)
