# Supplementary material for: Efficacy of house dust mite allergen immunotherapy in allergic rhinitis: a network meta-analysis based on component-resolved classification
Source: Front Immunol. 2026 Sep 3;17:1947642. doi: 10.3389/fimmu.2026.1947642 (PMC13582266; doi:10.3389/fimmu.2026.1947642)
Supplement: Supplementary file 8 [file Table1.docx]

Table S1.Full search strategies across all queried electronic databases

| Database | Search Query (Date limits: Inception to February 20 2026) |
| --- | --- |
| PubMed | (rhin* OR rhinitis OR rhinoconjunctivitis) AND (immunotherapy OR desensitization OR immunologic* OR SCIT OR SLIT) AND (mite* OR perennial OR HDM OR dermatophagoides OR "house dust") AND (controlled OR randomized OR placebo OR trial OR randomly OR blind*) |
| Embase | ('allergic rhinitis'/exp OR rhin*:ab,ti) AND ('immunotherapy'/exp OR immunotherapy:ab,ti OR desensiti*:ab,ti OR hyposensiti*:ab,ti OR AIT:ab,ti OR SIT:ab,ti OR SLIT:ab,ti OR SCIT:ab,ti) AND ('mite'/exp OR mite*:ab,ti OR 'house dust mite*':ab,ti OR HDM:ab,ti OR dermatophagoides:ab,ti OR perennial:ab,ti) AND ('randomized controlled trial'/exp OR 'controlled clinical trial'/exp OR randomized:ab,ti OR placebo:ab,ti OR randomly:ab,ti OR trial:ab,ti) |
| Cochrane Library (CENTRAL) | [mh "Rhinitis, Allergic"] OR (rhinitis:ti,ab,kw) OR (rhin*:ti,ab,kw) AND [mh Immunotherapy] OR (immunotherapy:ti,ab,kw) OR (desensiti*:ti,ab,kw) OR (hyposensiti*:ti,ab,kw) OR (AIT:ti,ab,kw) OR (SIT:ti,ab,kw) OR (SLIT:ti,ab,kw) OR (SCIT:ti,ab,kw) AND [mh Mites] OR (mite*:ti,ab,kw) OR (house NEXT dust NEXT mite*:ti,ab,kw) OR (HDM:ti,ab,kw) OR (Dermatophagoides:ti,ab,kw) OR (perennial:ti,ab,kw) |
