# Supplementary material for: Efficacy of house dust mite allergen immunotherapy in allergic rhinitis: a network meta-analysis based on component-resolved classification
Source: Front Immunol. 2026 Sep 3;17:1947642. doi: 10.3389/fimmu.2026.1947642 (PMC13582266; doi:10.3389/fimmu.2026.1947642)
Supplement: Supplementary file 9 [file Table2.docx]

**Table S2.Model fit and effect size comparisons between random-effects and fixed-effects models**

| Outcome | Model | DIC | CC-SCIT | MCD-SCIT | MCD-SLIT-tablet |
| --- | --- | --- | --- | --- | --- |
| SS | Fixed-effects | 36.06 | −0.35 (−0.58, −0.12) | −0.24 (−0.51, 0.03) | −0.26 (−0.31, −0.22) |
| SS | Random-effects | 30.89 | −0.40 (−0.73, −0.11) | −0.27 (−0.59, 0.03) | −0.30 (−0.41, −0.20) |
| MS | Fixed-effects | 47.63 | −0.37 (−0.62, −0.12) | −0.32 (−0.59, −0.05) | −0.18 (−0.22, −0.13) |
| MS | Random-effects | 28.50 | −0.40 (−0.81, 0.00) | −0.38 (−0.72, −0.05) | −0.15 (−0.28, −0.01) |
| CSMS | Fixed-effects | 43.90 | −0.56 (−0.81, −0.32) | 0.15 (−0.18, 0.49) | −0.25 (−0.30, −0.20) |
| CSMS | Random-effects | 34.09 | −0.84 (−1.53, −0.37) | 0.12 (−0.42, 0.64) | −0.27 (−0.48, −0.08) |

DIC, deviance information criterion; SS, symptom score; MS, medication score; CSMS, combined symptom and medication score; CC-SCIT, comprehensive component subcutaneous immunotherapy; MCD-SCIT, major component driven subcutaneous immunotherapy; MCD-SLIT-tablet, major component driven sublingual immunotherapy tablet. Effect size values are expressed as standardized mean differences followed by their corresponding ninety-five percent credible intervals.
