# Supplementary material for: Efficacy of house dust mite allergen immunotherapy in allergic rhinitis: a network meta-analysis based on component-resolved classification
Source: Front Immunol. 2026 Sep 3;17:1947642. doi: 10.3389/fimmu.2026.1947642 (PMC13582266; doi:10.3389/fimmu.2026.1947642)
Supplement: Supplementary file 10 [file Table3.docx]

**Table S3. Sensitivity analysis using alternative prior distributions**

| Outcome | Treatment | Uniform_Prior_SMD | HalfNormal_Prior_SMD | Absolute_Mean_Diff |
| --- | --- | --- | --- | --- |
| SS | CC-SCIT | −0.40 (−0.73, −0.11) | −0.40 (−0.73, −0.11) | 0 |
|  | MCD-SCIT | −0.27 (−0.58, 0.03) | −0.27 (−0.58, 0.03) | 0.001 |
|  | MCD-SLIT-T | −0.30 (−0.41, −0.20) | −0.30 (−0.41, −0.20) | 0 |
| MS | CC-SCIT | −0.40 (−0.81, −0.01) | −0.40 (−0.81, 0.00) | 0.001 |
|  | MCD-SCIT | −0.38 (−0.73, −0.05) | −0.38 (−0.72, −0.05) | 0.001 |
|  | MCD-SLIT-T | −0.15 (−0.28, −0.01) | −0.15 (−0.28, −0.01) | 0 |
| CSMS | CC-SCIT | −0.83 (−1.52, −0.37) | −0.83 (−1.51, −0.37) | 0.005 |
|  | MCD-SCIT | 0.12 (−0.42, 0.64) | 0.12 (−0.41, 0.63) | 0.002 |
|  | MCD-SLIT-T | −0.27 (−0.48, −0.08) | −0.27 (−0.47, −0.08) | 0 |

SMD, standardized mean difference; SS, symptom score; MS, medication score; CSMS, combined symptom and medication score; CC-SCIT, comprehensive component subcutaneous immunotherapy; MCD-SCIT, major component driven subcutaneous immunotherapy; MCD-SLIT-T, major component driven sublingual immunotherapy tablet. Effect size values are expressed as standardized mean differences followed by their corresponding ninety-five percent credible intervals. Absolute mean differences represent the marginal numeric variations between the two distinct prior distribution models.
