# Supplementary material for: Efficacy of house dust mite allergen immunotherapy in allergic rhinitis: a network meta-analysis based on component-resolved classification
Source: Front Immunol. 2026 Sep 3;17:1947642. doi: 10.3389/fimmu.2026.1947642 (PMC13582266; doi:10.3389/fimmu.2026.1947642)
Supplement: Supplementary file 11 [file Table4.docx]

**Table S4. SUCRA values and posterior ranking uncertainty for each treatment and outcome**

| Outcome | Treatment | SUCRA (%) | Probability of ranking first (%) | Posterior mean rank | 95% credible rank interval |
| --- | --- | --- | --- | --- | --- |
| SS | CC-SCIT | 82.6 | 63.3 | 1.52 | 1-3 |
| SS | MCD-SCIT | 55.2 | 21.2 | 2.34 | 1-4 |
| SS | MCD-SLIT-T | 60.7 | 15.5 | 2.18 | 1-3 |
| SS | Placebo | 1.5 | 0.0 | 3.96 | 3-4 |
| MS | CC-SCIT | 79.8 | 52.5 | 1.61 | 1-3 |
| MS | MCD-SCIT | 78.5 | 46.0 | 1.65 | 1-3 |
| MS | MCD-SLIT-T | 39.9 | 1.5 | 2.80 | 2-3 |
| MS | Placebo | 1.9 | 0.0 | 3.94 | 3-4 |
| CSMS | CC-SCIT | 99.5 | 98.7 | 1.02 | 1-1 |
| CSMS | MCD-SCIT | 12.6 | 0.3 | 3.62 | 2-4 |
| CSMS | MCD-SLIT-T | 64.3 | 1.0 | 2.07 | 2-3 |
| CSMS | Placebo | 23.6 | 0.0 | 3.29 | 3-4 |

SUCRA, surface under the cumulative ranking curve; SS, symptom score; MS, medication score; CSMS, combined symptom and medication score; CC-SCIT, comprehensive-component subcutaneous immunotherapy; MCD-SCIT, major-component-dominant subcutaneous immunotherapy; MCD-SLIT-T, major-component-dominant sublingual immunotherapy tablet. Rank 1 indicates the most favorable treatment. The probability of ranking first, posterior mean rank, and the 95% credible rank interval were derived from the posterior rank distribution; the interval corresponds to the 2.5th and 97.5th percentiles. SUCRA and posterior ranking measures should be interpreted together with treatment-effect estimates, credible intervals, and the amount of evidence supporting each treatment node.
